# The effect of unhealthy *β*-cells on insulin secretion in pancreatic islets

**DOI:** 10.1186/1755-8794-6-S3-S6

**Published:** 2013-11-11

**Authors:** Yang Pu, Saangho Lee, David C Samuels, Layne T Watson, Yang Cao

**Affiliations:** 1 Department of Computer Science, Virginia Polytechnic Institute and State University, Blacksburg, Virginia 24061, USA; 2 Center for Human Genetics Research, Vanderbilt University, Nashville, Tennessee 37232, USA; 3 Department of Mathematics, Virginia Polytechnic Institute and State University, Blacksburg, Virginia 24061, USA

## Abstract

**Background:**

Insulin secreted by pancreatic islet *β*-cells is the principal regulating hormone of glucose metabolism and plays a key role in controlling glucose level in blood. Impairment of the pancreatic islet function may cause glucose to accumulate in blood, and result in diabetes mellitus. Recent studies have shown that mitochondrial dysfunction has a strong negative effect on insulin secretion.

**Methods:**

In order to study the cause of dysfunction of pancreatic islets, a multiple cell model containing healthy and unhealthy cells is proposed based on an existing single cell model. A parameter that represents the function of mitochondria is modified for unhealthy cells. A 3-D hexagonal lattice structure is used to model the spatial differences among *β*-cells in a pancreatic islet. The *β*-cells in the model are connected through direct electrical connections between neighboring *β*-cells.

**Results:**

The simulation results show that the low ratio of total mitochondrial volume over cytoplasm volume per *β*-cell is a main reason that causes some mitochondria to lose their function. The results also show that the overall insulin secretion will be seriously disrupted when more than 15% of the *β*-cells in pancreatic islets become unhealthy.

**Conclusion:**

Analysis of the model shows that the insulin secretion can be reinstated by increasing the glucokinase level. This new discovery sheds light on antidiabetic medication.

## Background

With 25-30 percent of adults in the developed world having a high risk of diabetes, and 24 million people in the United States with diabetes, diabetes mellitus ranks as a principal cause of death [1]. The incidence of diabetes mellitus is expected to double within the coming 20 years. After a meal, healthy individuals secrete insulin into the bloodstream signaling the consumption of glucose produced from the digested food. Thus insulin has an important signaling role in the maintenance of low blood glucose levels via glucose consumption. Type II diabetes may result from disruption of this signaling process [2,3]. Hence understanding the insulin secretion mechanism is vitally important.

**Figure 1:**
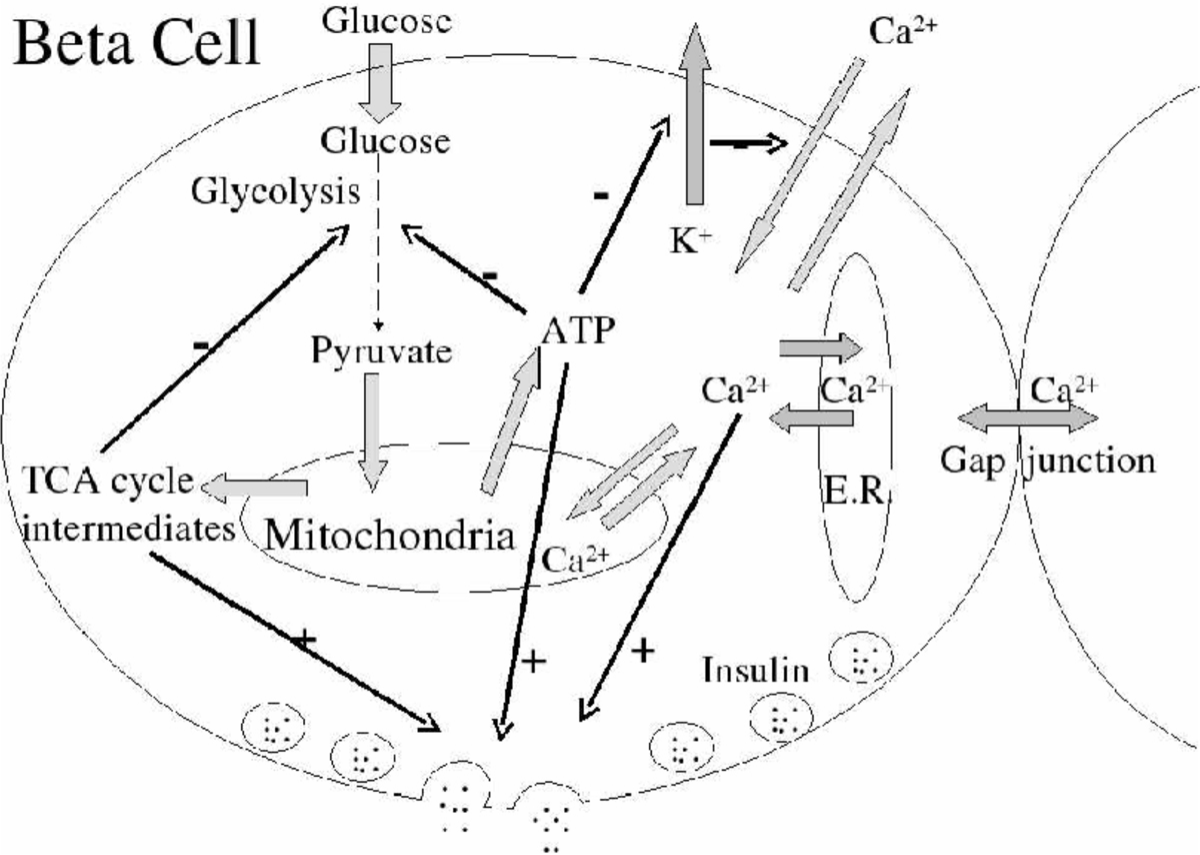
**Metabolic pathway in the *β*-cell**.

The main pathways involved in glucose-stimulated insulin secretion are shown in Figure 1. Glucose is imported into *β*-cells and undergoes the glycolysis process. Three primary products, pyruvate, malate, and nicotinamide adenine dinucleotide plus hydrogen (NADH), are produced in glycolysis [4] and transported into mitochondria. In mitochondria pyruvate and malate are consumed in the tricarboxylic acid (TCA) cycle to produce fumarate. Adenosine triphosphate (ATP), the most important energy resource in the cell, is produced from fumarate and NADH through oxidative phosphorylation and released to the cytoplasm [5]. The increasing ATP level in the cytoplasm blocks the ATP-sensitive K^+ ^channel [6-8], resulting in a higher intracellular *K*^+ ^level and depolarization of the cell membrane. This results in the opening of voltage-dependent Ca^2+ ^channels. Cytoplasmic Ca^2+ ^is transported into the *β*-cell from the extracellular fluid through the voltage-gated Ca^2+ ^channels [9-11]. When both the cytoplasmic ATP and Ca^2+ ^levels rise high enough, the vesicles containing insulin fuse with the cell plasma membrane, releasing insulin from the cell. The increasing formation and export of ATP to the entire cell will raise the Ca^2+ ^levels in the mitochondria as well. High mitochondrial Ca^2+ ^levels eventually collapse the mitochondrial membrane potential, shutting off ATP production, possibly by opening a low-conductance state of the permeability transition pore (PTP) in the mitochondrial membrane [12-14]. The rise and fall in the mitochondrial membrane potential and intramitochondrial Ca^2+ ^concentration is the mitochondrial oscillation. The frequency of this oscillation depends on how rapidly the mitochondria can build up its membrane potential and transport Ca^2+^. Besides the metabolic pathway in a single *β*-cell, cells in each pancreatic islet are also connected through direct electrical connections between neighboring *β*-cells. This connection is sufficient to synchronize both electrical bursting activity and metabolic oscillations [15].

The cells, in fact, have differing capabilities in consuming glucose, since their differing physical properties cause different rates of electron transport chain function, ATP production, Ca^2+ ^uptake and release, and cell membrane potential. Furthermore, some *β*-cells do not function normally as just described. The term *unhealthy *is used to describe cells that cannot consume glucose normally. Unhealthy cells interact strongly with adjacent healthy cells, because of their electrical connections. The total insulin secretion is the aggregation of the insulin secreted by all pancreatic islet cells. Its dynamics may be destroyed by a small fraction of unhealthy cells even though a large majority of the cells are still healthy. Consequently, understanding how a cohort of unhealthy cells can affect the insulin secretion process in other healthy cells is very important, and can lead to a better understanding of the cause of diabetes.

This present work proposes a mathematical model to study scenarios with unhealthy cells leading to insulin oscillation failure. The new model is based on a mathematical insulin secretion model of the oscillation and metabolic pathway of insulin secretion in a single *β*-cell, due to Bertram et al. [16-18]. Bertram's original model is extended to model spatial and coupling effects [14] due to the electrical connections between neighboring *β*-cells. Mitochondrial dysfunction is believed to have a strong negative effect on insulin secretion [19-21], and also mitochondrial dysfunction is believed to be a main cause of unhealthy cells, based on the recent data of [22-24]. The ratio of the total mitochondrial volume to the cytoplasm volume per *β*-cell, a parameter corresponding to mitochondria function, is used to define unhealthy cells, as distinguished from healthy cells. The effect on the islet insulin oscillation of unhealthy cells coupled with healthy cells is then studied in detail. Multiple cell simulations demonstrate insulin oscillation malfunction when the fraction of unhealthy cells exceeds approximately 15%. To verify this result, a simplified coupling topology is used to study the effect of one unhealthy cell on neighboring healthy cells. The latter study confirmed the previous more complicated simulation.

In order to understand the cause of insulin secretion failure resulted from unhealthy cells, an eight-cell model is built and studied in details. We discovered a critical difference of the dynamics between healthy cells and unhealthy cells. Our simulation results demonstrate that stimulating glucokinase can make unhealthy pancreatic islets function normally. Based on the discovery, a possible strategy for antidiabetic medicine is proposed. Our strategy is consistent with recent antidiabetic medicine development [25-30] that identifies glucokinase as a major drug target.

## Methods

### Single *β*-cell modeling

**Figure 2:**
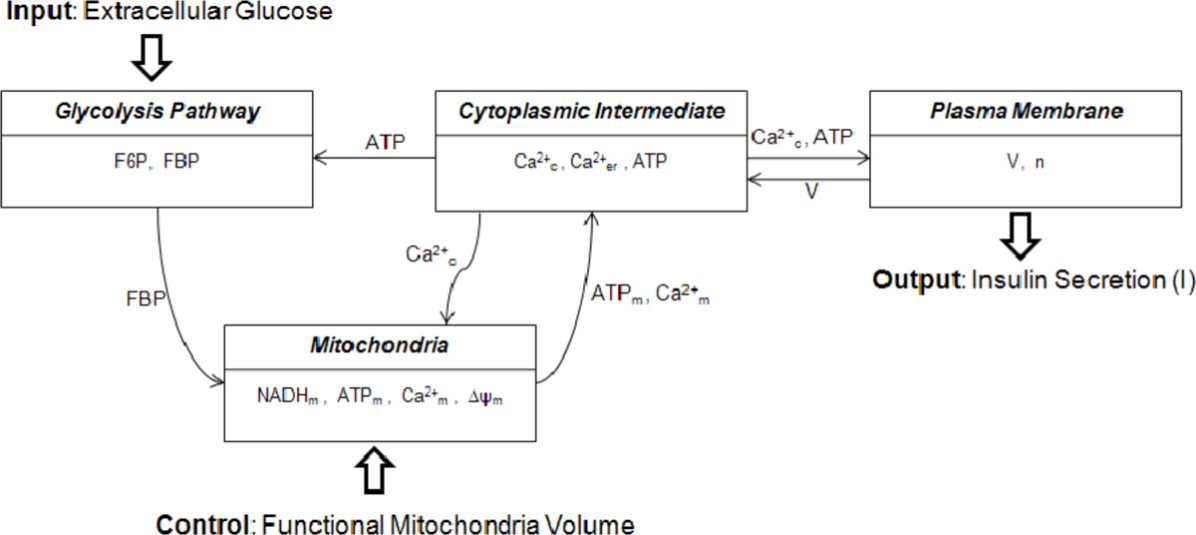
**Model components and interconnections**. The whole model is divided into four components: glycolytic, mitochondrial metabolism, cytoplasmic intermediate, and plasma membrane.

The mathematical model of a single pancreatic *β*-cell is based on the deterministic model introduced by Bertram et al. [16-18]. The model has four components: glycolytic, mitochondrial metabolism, cytoplasmic intermediate, and plasma membrane. Each component of the model is connected by variables shared between components as illustrated in Figure 2.

The glycolytic component of the model is a simplified model of the glycolysis pathway [31] that converts glucose into pyruvate. This pathway generates oscillations via a critical enzyme phosphofructokinase (PFK), the rate limiting variable in glycolysis and an important control. PFK is allosterically inhibited by ATP [32]. As a result high ATP levels will cause glycolysis to slow down. PFK phosphorylates fructose-6-phosphate (F6P) to fructose-1,6-bisphosphate (FBP), which is a substrate for glyceradehyde 3-P dehydrogenase (GPDH). The GPDH reaction is in a rapid equilibrium, therefore used as the input to the mitochondrial component, replacing pyruvate dehydrogenase (PDH) in the model. The glycolysis model consists of equations for the concentrations [G6P] and [FBP]. Here [G6P] is the glucose 6-phosphate concentration, which is assumed to be in rapid equilibrium with [F6P], and the *J*s are fluxes.

(1a) $$\frac{d\left[G6P\right]}{dt}=J_{GK}-J_{PFK},$$

(1b) $$\frac{d\left[FBP\right]}{dt}=J_{PFK}-\frac{1}{2}J_{GPDH}.$$

The model for the mitochondrial component is based on the detailed Magnus-Keizer model of mitochondrial metabolism [33]. The tricarboxylic acid cycle (TCA cycle) and oxidative phosphorylation occur in mitochondria. The TCA cycle is a series of enzyme-catalyzed chemical reactions whose main role is to convert pyruvate to NADH and reduced flavin adenine dinucleotide (FADH), and then feed them into oxidative phosphorylation, which uses energy released by the oxidation of nutrients to produce ATP [34]. The model includes an expression for Ca^2+^-dependent dehydrogenases of the citric acid cycle, yielding NADH. The NADH supplies electrons for the electron transport chain, which produces a membrane potential Δ*ψ *across the mitochondrial inner membrane. The flux of protons down the electrical gradient through the F1F0-ATP synthase converts ADP to ATP. Finally, Ca^2+ ^enters the mitochondria through Ca^2+ ^uniporters and is transported out through Na^+^/Ca^2+ ^exchangers [35]. The mitochondrial compartment has variables for the NADH, ADP, and mitochondrial Ca^2+ ^concentrations, and for Δ*ψ *The ATP concentration is calculated from the ADP concentration by assuming that the sum of the two is conserved. Differential equations for the mitochondrial variables are

(2a) $$\frac{d\left[NADH_{m}\right]}{dt}=\gamma (J_{DH}-J_{O}),$$

(2b) $$\frac{d\;\Delta \psi }{dt}=\left(J_{H,Res}-J_{H,atp}-J_{ANT}-J_{H,leak}-{J_{Na}}_{Ca}-2J_{uni}\right)/C_{m},$$

(2c) $$\frac{d\left[Ca_{m}\right]}{dt}=f_{m}\left(J_{uni}-{J_{Na}}_{Ca}\right),$$

(2d) $$\frac{d\left[ADP_{m}\right]}{dt}=\gamma \left(J_{ANT}-J_{F1F0}\right).$$

There are five variables in the cytoplasmic intermediate and plasma membrane components, which describe the plasma membrane potential *V *and the cytosolic ADP concentration, the fraction *n *of the opened delayed rectifying K^+ ^channels and cytosolic Ca^2+ ^concentration, as well as the ER Ca^2+ ^concentration. The differential equations are

(3a) $$\frac{dV}{dt}=-\left(I_{K}+I_{Ca}+I_{K\left(Ca\right)}+I_{K\left(ATP\right)}\right)/C,$$

(3b) $$\frac{dn}{dt}=\frac{n_{\infty }\left(V\right)-n}{\tau },$$

(3c) $$\frac{d\left[Ca_{c}\right]}{dt}=f_{c}\left(J_{mem}+J_{er}+\kappa J_{m}\right),$$

(3d) $$\frac{d\left[Ca_{er}\right]}{dt}=-f_{er}\left(V_{c}/V_{er}\right)J_{er},$$

(3e) $$\frac{d\left[ADP_{c}\right]}{dt}=J_{hyd}-\kappa J_{ANT},$$

where *I_K_*, *I_Ca_*, *I*_*K*(*Ca*)_, and *I*_*K*(*ATP*) _are the ionic current on the membrane, *C *is the membrane capacitance, *f_c _*is the fraction of free Ca^2+ ^in the cytosol, *J_mem _*is the flux of Ca^2+ ^across the plasma membrane, *J_m _*is the flux of Ca^2+ ^out of the mitochondria, which is scaled by the mitochondria/cytosol volume ratio *κ*, *J_er _*is the flux out of the ER, *f_er _*is the fraction of free Ca^2+ ^in the ER, *V_c _*and *V_er _*are the volumes of the cytosolic and ER compartments, respectively, *n *is the fraction of the open delayed rectifying K^+ ^channels, *τ *is the relaxation time for the open and close reactions of the delayed rectifying K^+ ^channels to reach equilibrium, and the steady state function for *n *is *n*_∞_(*V*) = (1 + exp(−(*V *+ 16)*/*5))^−1^. For more details on these terms in the differential equations of the model, refer to Bertram et al.

### Multiple *β*-cells modeling

**Figure 3:**
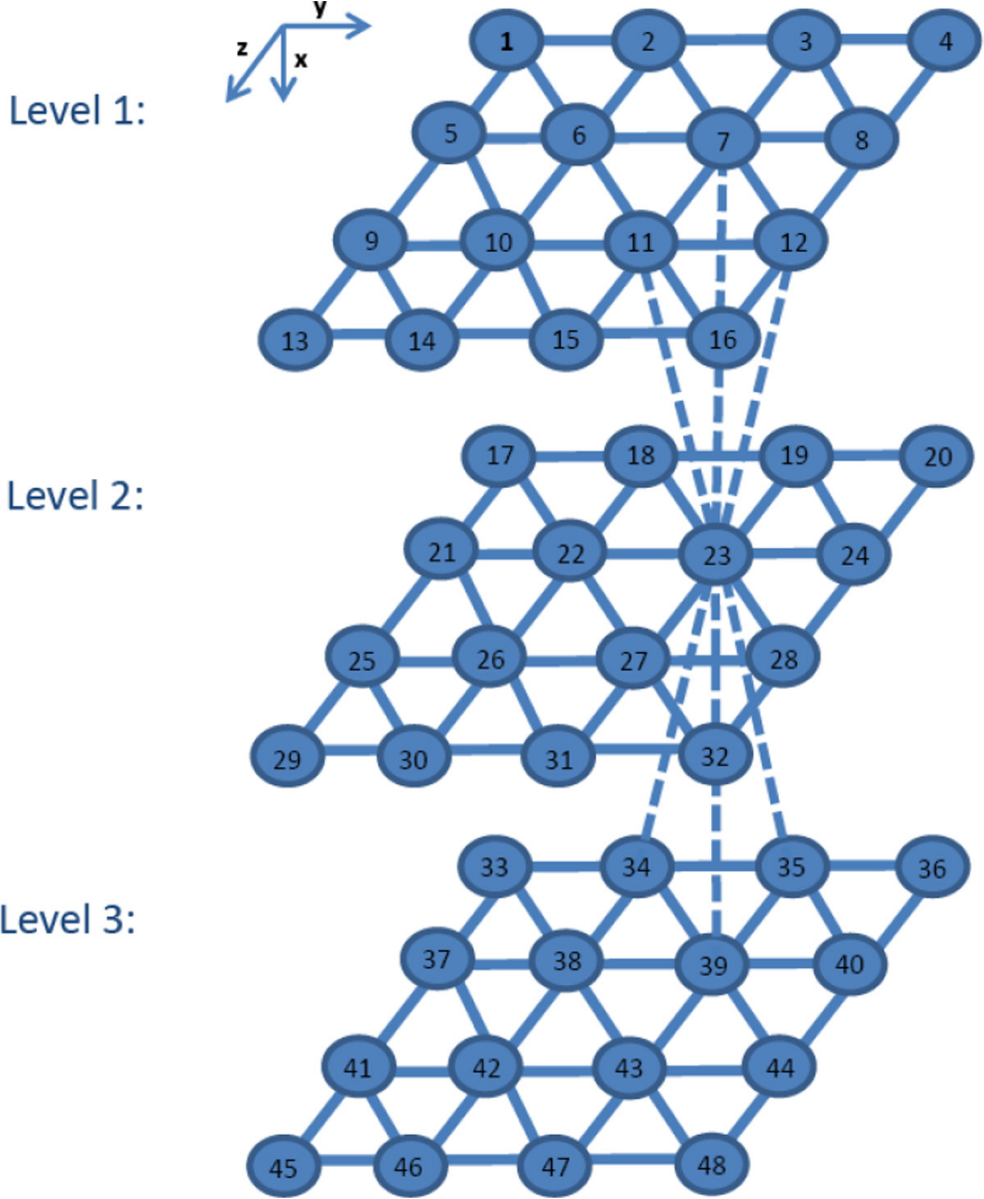
**Three layers of *β*-cells in 3-D hexagonal lattice**.

There are about 1, 000 *β*-cells in each pancreatic islet. They interact with neighboring *β*-cells through direct electrical connections. These interactions are modeled with a change in (3a):

(4) $$\begin{array}{c} \;\;\;\;\;\;\;\;\;\;\;\;\;\;\;\;\;\;\;\;\;\;\;\;\;\;\;\;\;\frac{dV_{j}}{dt}=-\left(I_{K}+I_{Ca}+I_{K\left(Ca\right)}+I_{K\left(ATP\right)}+I_{j}\right)/C,\\ I_{j}=gc\underset{i\in N\left(j\right)}{\sum }\left(V_{j}-V_{i}\right), \end{array}$$

where *I_j _*is the current for cell *j*, *gc *is the electrical coupling conductance, and *N *(*j*) is the index set for all neighbor cells of cell *j*. The neighbors surrounding a cell *j *are detected by considering the position of a cell *j *within a 3-D hexagonal lattice (different from the 3-D von Neumann neighborhoods of cellular automata theory). As shown in Figure 3, each internal cell is connected with six cells in the same layer, three cells in the upper layer and three other cells in the lower layer. For example, cell 23 in Figure 3 is connected with cells 18, 19, 22, 24, 27, 28 in its own layer, and connected with cells 7, 11, 12 in the upper layer, and cells 34, 35, 39 in the lower layer. Face, edge, and corner cells have between three and nine neighbors, depending on their location. Table 1 is used to calculate the coordinates of the neighbors of cell (*a, b, c*) in the 3-D hexagonal lattice.

**Table 1:** The coordinates of the neighbors of the cell with coordinates (a, b, c)

Layer	Coordinates (*x*, *y*, *z*)
	(*a − *1*, b, c*)
Upper Layer	(*a − *1*, b *+ 1*, c *+ 1)
	(*a − *1*, b, c *+ 1)

	(*a*, *b*, *c − *1)
	(*a*, *b*, *c *+ 1)
Same Layer	(*a*, *b − *1*, c *− 1)
	(*a*, *b − *1*, c*)
	(*a*, *b *+ 1*, c*)
	(*a*, *b *+ 1*, c *+ 1)

	(*a *+ 1, *b *− 1, *c − *1)
Lower Layer	(*a *+ 1*, b, c − *1)
	(*a *+ 1*, b, c*)

Besides the spatial differences, each cell has its own parameter set. Among all the parameters of the single cell model, *κ*, the ratio of mitochondria volume to cytosol volume, represents the function of mitochondria. The single cell study has shown that the oscillation behavior of a cell is very sensitive to *κ*, and thus *κ *is used to describe the cell differences. The value of *κ *is different for each cell in the 3-D structure, while all other cell parameters are set the same.

## Results and discussion

The differential equations for the model, implemented in Fortran 90, were solved using LSODE in ODEPACK [36]. Generated data were plotted using Matlab. The simulations are performed on a Mac OS 2.4 GHz Intel(R) Core 2 Duo CPU with 4GB memory.

### Single *β*-cell simulation

The simulation model for multiple cells has at its core the single cell model. Since the total amount of ATP and ADP (in the single cell model) is conserved, only ADP appears in the differential equation

(5) $$\frac{d\left[ADP_{c}\right]}{dt}=J_{hyd}-\kappa J_{ANT},$$

where *J_hyd _*is the hydrolysis rate of ATP to ADP, *κ *(*κ *= 0.0733 in the standard model [37]) is the ratio of the total functional mitochondrial volume to the cytoplasm volume, and *J_ANT _*is the flux through the adenine nucleotide translocator, which exchanges ADP and ATP molecules between the cytoplasm and the mitochondria. With *κ *= 0.0733, both the cell membrane potential and insulin secretion show periodic bursts of rapid oscillation, as illustrated in Figure 1 of Additional file 1. The slowest component of the compound bursting is due to oscillatory glycolysis, reflected by an oscillatory FBP concentration. The oscillatory glycolysis causes slow oscillations in ADP, which superimpose with the faster ADP oscillations driven by Ca^2+^. The multimodal ADP rhythm leads to oscillations in the conductance of the ATP-dependent potassium channel, which drives the burst episodes of the membrane potential *V*, which then results in compound bursting of intracellular calcium, leading to pulsatile insulin secretion.

In truth, though, *κ *varies between cells; specifically, cells with some mitochondria weakened due to aging will effectively have a *κ *smaller than the default value. It is therefore paramount to study the sensitivity of the cell membrane potential *V *and insulin secretion *I *patterns to the parameter *κ*. For this work, Bertram's single cell model is modified by varying the parameter *κ *to represent different levels of functional mitochondria within different cells. In terms of changes in the patterns of *V*, Ca^2+^, and *I*, the bursting behavior of the model is classified into four categories as a function of the volume of functioning mitochondria in the *β*-cell, in order of occurrence: burst formation, periodic burst, burst loss, and decoupling.

The bursting pattern starts to form at around 85% of the regular mitochondrial volume, which is when the total volume of the mitochondria is about 6% of the cytoplasmic volume. If the volume of functioning mitochondria is lower than this limit, insufficient ATP is produced to allow the insulin burst. In the burst formation category (Additional file 1: Figure 2) each burst consists of a small number of spikes in plasma membrane voltage, cytoplasmic calcium ion concentration, and the insulin secretion rate. If the mitochondrial volume fraction is increased beyond the value used in the standard model, the behavior of the burst begins to change in pattern, entering the burst loss category (Additional file 1: Figure 3). In the burst loss category every other burst decreases in duration until it is lost completely, while the remaining bursts lengthen their duration. The net effect is a decrease in the fraction of time spent in each burst.

When the volume fraction of the mitochondria rises over about 0.095 the oscillation patterns undergo a radical alteration (Additional file 1: Figure 4). The frequency of the cell membrane voltage oscillation within the bursts becomes very large. While the membrane voltage continues to undergo bursts of rapid oscillations separated by periods of no oscillation, this no longer drives the calcium ion concentration, which then normally drives the insulin secretion. Thus this behavior will be called the decoupling category. While insulin release does undergo a long and slow periodic variation, the rapid spikes that characterize the normal burst no longer occur.

**Figure 4:**
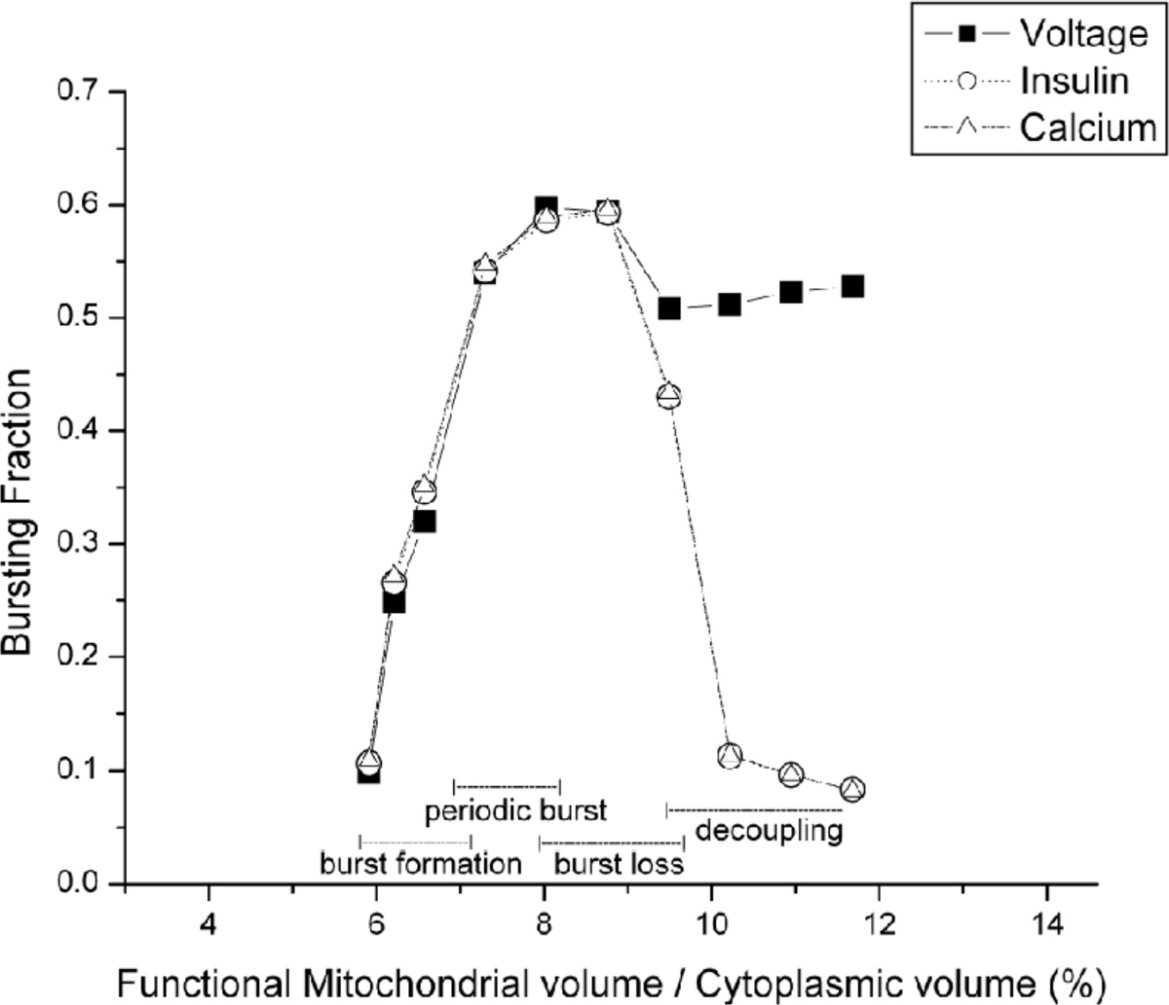
**Bursting fraction for the cell membrane potential *V *, the calcium ion concentration *Ca*^2+^, and the insulin secretion rate *I*.** The X-axis represents the ratio of functional mitochondrial volume and the cytoplasmic volume. The Y-axis represents the bursting fraction.

In order to quantify the development and eventual loss of the bursting behavior, define the "bursting fraction" as the portion of the total time taken up by the bursts, where a burst duration is defined as the time from the first to the last peak in a burst. Bertram's single cell original model has a bursting fraction of approximately 0.55 (meaning that a burst in the model variables' oscillations is occurring for 55% of the time). When the cell mitochondrial volume fraction is changed, this bursting fraction changes markedly (cf. Figure 4), and the patterns of the calcium ion concentration Ca^2+^, the cell membrane potential *V*, and the insulin secretion rate *I *all alter noticeably. Figure 4 shows the ranges of the mitochondrial volume ratios corresponding to the four classifications: burst formation, periodic bursts, burst loss, and decoupling. Since the classifications are qualitative, the divisions between the classifications are blurred and the ranges are drawn overlapping. The membrane voltage, calcium ion concentration, and insulin release all have nearly identical bursting fractions for the first three categories (formation, periodic busts, and loss). In the decoupling category the bursting fractions in the calcium ion concentration and insulin release rapidly fall to zero, while the voltage bursting fraction holds relatively constant. The normal bursting behavior of the single *β*-cell model occurs only within a narrow range of mitochondrial volumes, from roughly 7% to 8% of the cellular volume.

**Figure 5:**
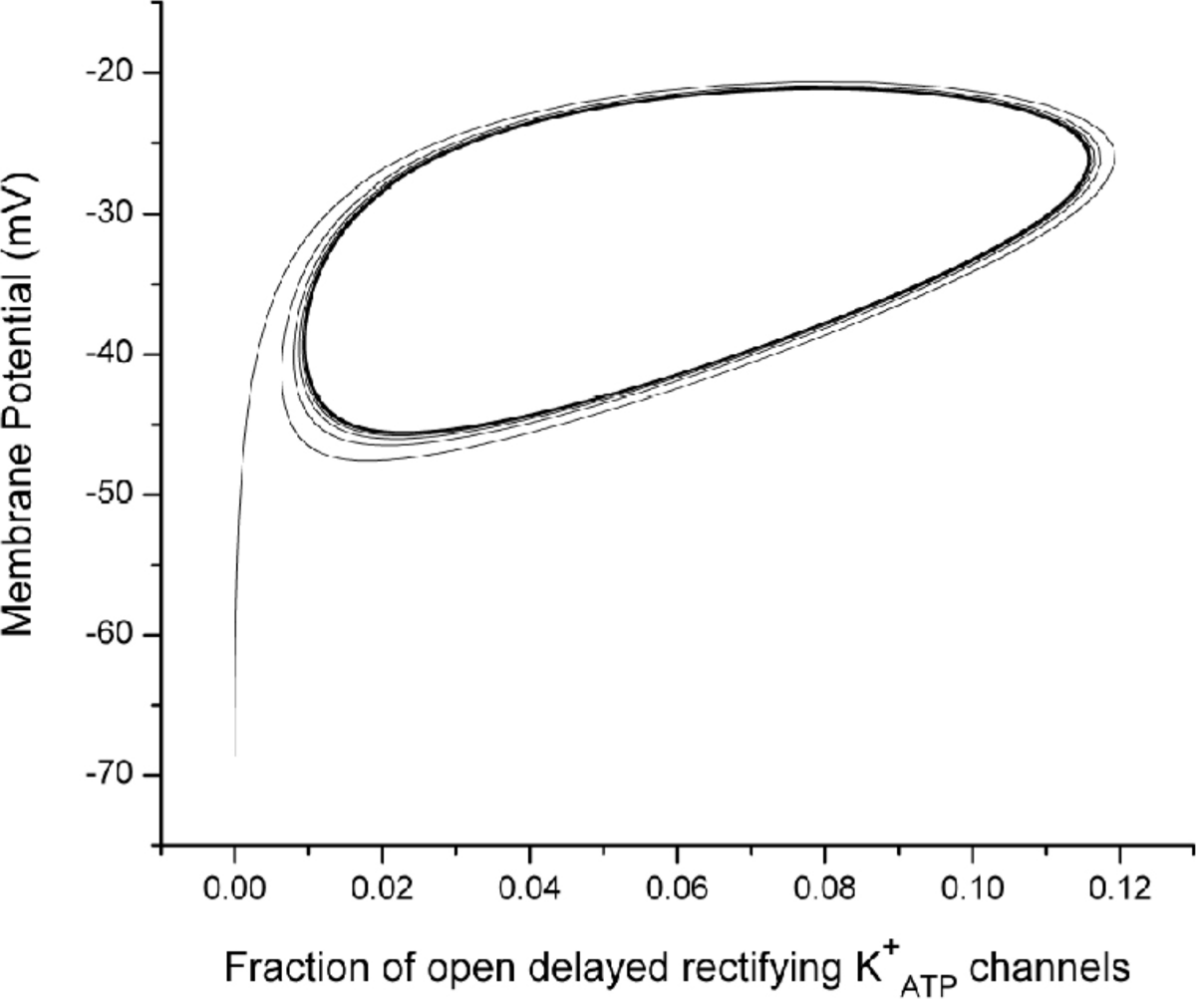
**Phase plane showing relationship between plasma membrane potential *V *an activation variable for the delayed rectifying *K*^+ ^channels *n*.** The X-axis represents the fraction of open delayed rectifying $K_{ATP}^{+}$ channels. The Y-axis represents the membrane potential with a unit of *mV *.

The reason for the absence of the bursting function for low mitochondrial volume is straightforward. With a lower volume of functioning mitochondria the cytoplasmic ATP levels are lower and are insufficient to trigger the remaining stages of the signaling pathway for insulin release (Figure 1). The change in behavior at higher mitochondrial volumes, where cytoplasmic ATP levels are slightly higher, has a more complex mechanism. This model of the insulin secretion is built around a fast central oscillator [10,38,39] consisting of the plasma membrane potential *V *and the fraction *n *of open delayed rectifying potassium ion channels, denoted by K^+ ^in Figure 1. As the symbol implies, the opening and closing of these potassium channels is controlled by the cytoplasmic ATP concentration. During the bursts these two variables execute a fast oscillation (Figure 5), which drives the other oscillations of this model system. Between bursts, this oscillation stops and the *V *and *n *values move over to the "tail of the Q" in Figure 4, until the next burst begins. The period of this central oscillator driving the whole model is controlled by the ATP dependence on the opening of the potassium channels. Higher ATP levels lead to a faster oscillation of *V *and *n*, which (in this model) becomes too fast for the dynamics of the cytoplasmic calcium ion concentration to follow, decoupling the rest of the system from the central oscillator. Therefore, altering the mitochondrial/cytosol volume ratio in the model, which corresponds to the amount of functional mitochondria in the cell, alters the amount of ATP production and through this the level and pattern of insulin secretion. In order to reflect cell differences, the value of *κ *is different for each cell in the 3-D structure, with all other cell parameters being the same.

The insulin bursts are totally absent when the volume fraction of the mitochondria is either less than 0.06 or greater than 0.095, which suggests an alternative definition for "healthy cell" and "unhealthy cell". Define a *healthy cell *as having a mitochondria volume fraction *κ *between 0.06 and 0.095, and a cell with *κ *out of this range as an *unhealthy cell*. It is plausible that some mitochondria lose their function, effectively resulting in a smaller *κ*. The ensuing numerical experiments use *κ *= 0.05 for unhealthy cells and the standard value *κ *= 0.0733 for healthy cells.

### Multiple *β*-cells simulation

Although there are about 1,000 cells in each pancreatic islet, for multiple *β*-cells simulations, consider first the case with 125 cells coupled spatially in a 3-D hexagonal lattice. The justification for using 125 rather than 1000 cells is a pragmatic one--the CPU time for simulating 1,000 cells is rather long. In a 1,000 cell heterogeneous model, each single cell model has ten variables, yielding a 10,000-dimensional ODE, which required 26 hours to solve till time *t *= 2 × 10^6^. Furthermore, the oscillation patterns observed from 125 cells are qualitatively very similar to those observed from 1,000 cells.

If there are no unhealthy cells at all, the membrane potentials of all the cells synchronize after the coupling is turned on at time 400,000 milliseconds (ms) by changing *gc *from 0 to 150. This simulation of 125 cells without unhealthy cells is shown in Figures 1 (membrane potential) and 2 (total insulin secretion) in Additional file 2. The curves in the membrane potential plot are out of phase at time *t *= 0, but soon after 400,000 ms, these curves coalesce (see Additional file 2: Figure 1). Because the insulin levels of some cells are high while those of other cells are low, the total insulin is relatively flat before synchronization. Immediately after the coupling is turned on, the total insulin secretion shows bursts and its value rises to a hundred times that of a single cell, because there are more than a hundred cells synchronized and releasing insulin in phase.

Focus on total insulin secretion to see how unhealthy cells, through the 3-D coupling in the hexagon structure, affect the total insulin secretion. To save computational time the coupling is turned on at the beginning of the simulations, *t *= 0. Figure 3 in Additional file 2 shows the resulting total insulin behavior with 10% of the cells being unhealthy spread uniformly in the 3-D hexagonal structure. The total insulin, as in the case of 100% healthy cells, shows periodic oscillations and maintains a reasonable level. When the percent of cells being unhealthy increases to 15%, the oscillations of total insulin still look normal (see Additional file 2: Figure 4), but now some bursts have fewer spikes. As the percentage of cells being unhealthy increases to 20% and 30% from 10% and 15% of cells being unhealthy, the spikes within each burst become much less numerous (shown in Additional file 2: Figures 5 and 6). These bursts are also much more irregular, and even more significantly, totally disappear after 2.25 × 10^6 ^ms (Additional file 2: Figure 6). In summary, the cohort of unhealthy cells dominates the global behavior, resulting in a level of total insulin too low to maintain proper pancreatic islet function. The conclusion is that if there is more than approximately 15% of cells being unhealthy, the function of the pancreatic islet will be severely affected.

To verify the conclusions from simulations with 125 cells, simulations were also performed for the model with 1,000 cells. This 1000 cell model cannot be run to as long a time as the 125 cell model, but observe that when, for a certain time, insulin secretion cannot generate enough spikes, the pancreatic islet can be considered to be malfunctioning. Hence the simulation monitors the numbers of spikes in the last five bursts, and if the mean number of spikes is below three, deems that the overall system is malfunctioning and halts. The simulations for 1,000 cells with 30%, 20%, 15% of cells being unhealthy, all considered malfunctioning systems, are shown in Figures 7, 8, and 9 of Additional file 2 respectively. A simulation for 10% of cells being unhealthy found no malfunction in an extremely long time (comparable to the time for the 125 cell model runs). In summary, the 125 cell and 1000 cell simulations yield similar conclusions: If the percentage of cells being unhealthy is larger than approximately 15%, the system will malfunction; at 15% of cells being unhealthy, the system still functions but is close to malfunctioning; below 10% of cells being unhealthy, the system can definitely function very well.

Another ratio, the number of links between an unhealthy cell and a healthy cell to the total number of links between all cells, is also calculated for each simulation; the value of this ratio is quite close to the number of unhealthy cells to total number of cells ratio, see Table 2. Therefore, for further analysis, using 125 cells and the ratio of number of unhealthy cells to total number of cells should suffice.

**Table 2:** Comparison of Two Different Ratios

Ratio of Links	Ratio of Cells
0.0945	0.10
0.1477	0.15
0.1963	0.20
0.2994	0.30

### Simplified multiple *β*-cells simulation

**Figure 6:**
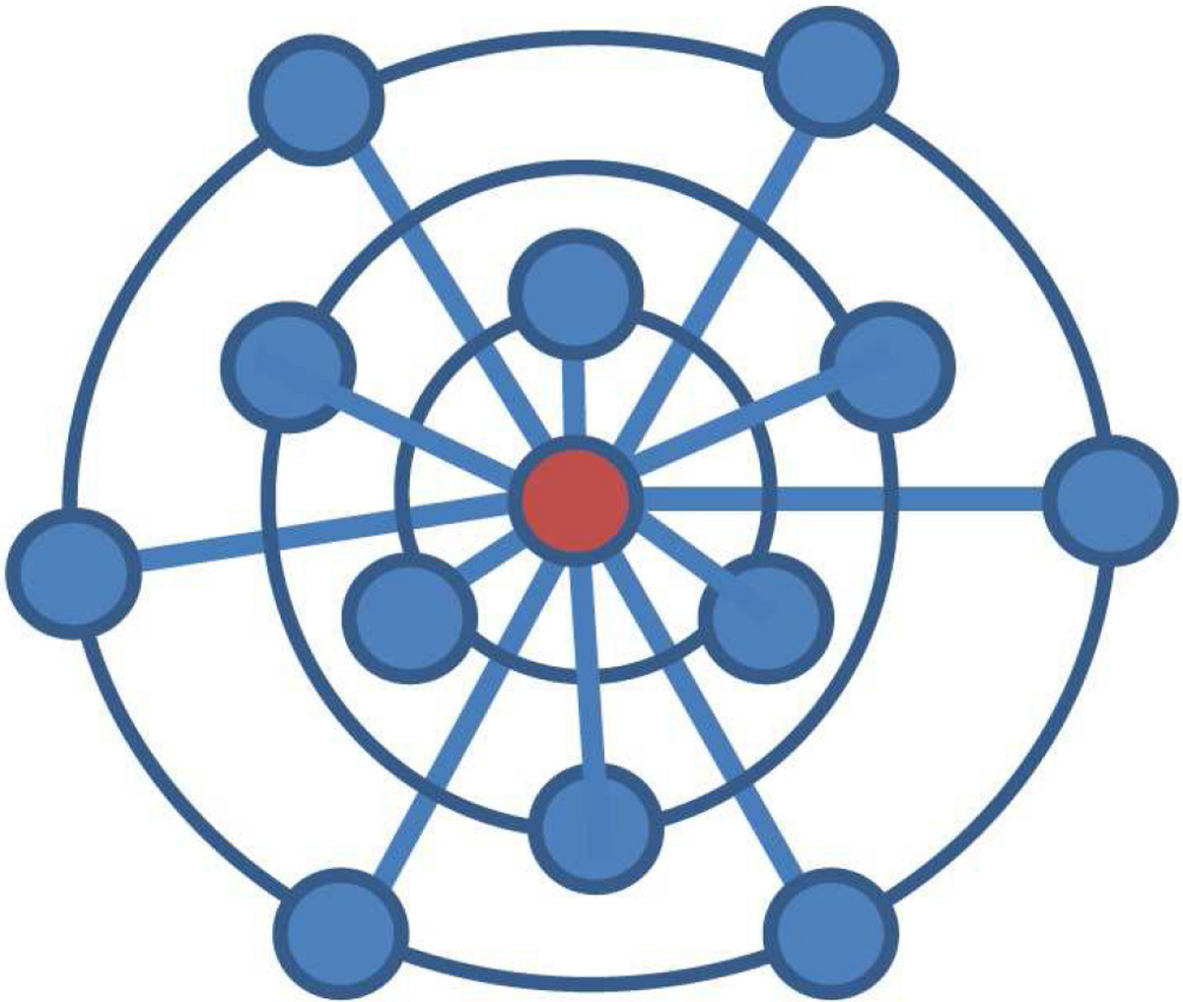
****Topology for one unhealthy cell****. A simplified case with only one unhealthy cell in the 3-D structure. The red dot (center dot) represents the unhealthy cell, the blue dots are healthy cells.

**Figure 7:**
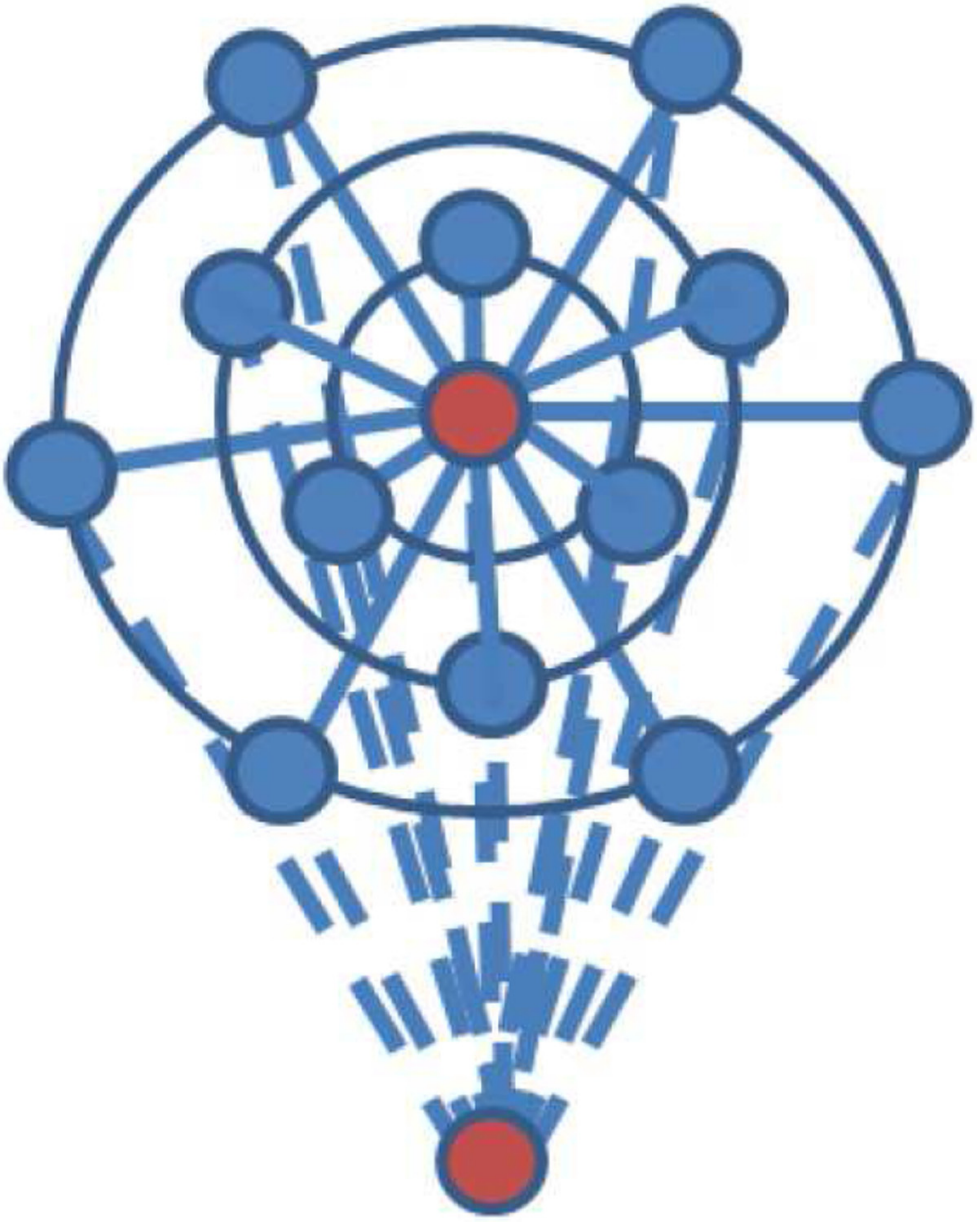
****Topology for two unhealthy cells****. The simplified topology for two adjacent unhealthy cells in two layers.

In order to understand further how unhealthy cells affect the healthy cells, consider a simplified case with only one unhealthy cell in the 3-D structure, as shown in Figure 6. The red dot represents the unhealthy cell, the blue dots healthy cells. The unhealthy cell is connected with all the healthy cells, while the healthy cells themselves are connected through three rings corresponding to three layers in the 3-D structure. The experiment starts with two cells, one healthy and one unhealthy. Then the number of healthy cells is increased one by one. If the total number of cells is smaller than 13, the corresponding subgraph of Figure 6 is used. Figure 1 in Additional file 3 shows the total insulin of one unhealthy cell and one healthy cell (coupling starts at 3 × 10^5 ^ms). According to the "malfunction criterion" (the average number of spikes in the last five bursts is smaller than three), the system malfunctions. Figure 2 in Additional file 3 shows the total insulin for one unhealthy cell and three healthy cells. The simulations show that when the number of healthy cells reaches three (marginally) or four, the total insulin shows functioning bursts. This suggests that one unhealthy cell needs at least four healthy cells to make the whole system function well based on the topology in Figure 6. To study the case of two unhealthy cells with exactly the same topology and connected to the same number of healthy cells, the topology in Figure 7 is used. The number of healthy cells needed to rescue two unhealthy cells is eight (marginally) or nine, which is consistent with the result of one unhealthy cell in Figure 6. Therefore one unhealthy cell needs at least four healthy cells to make the overall system function well, i.e., the system probably can tolerate at most 20% of cells being unhealthy. This result matches with the previous simulations with 125 and 1,000 cells in the 3-D topology.

## The cause of insulin secretion failure

**Figure 8:**
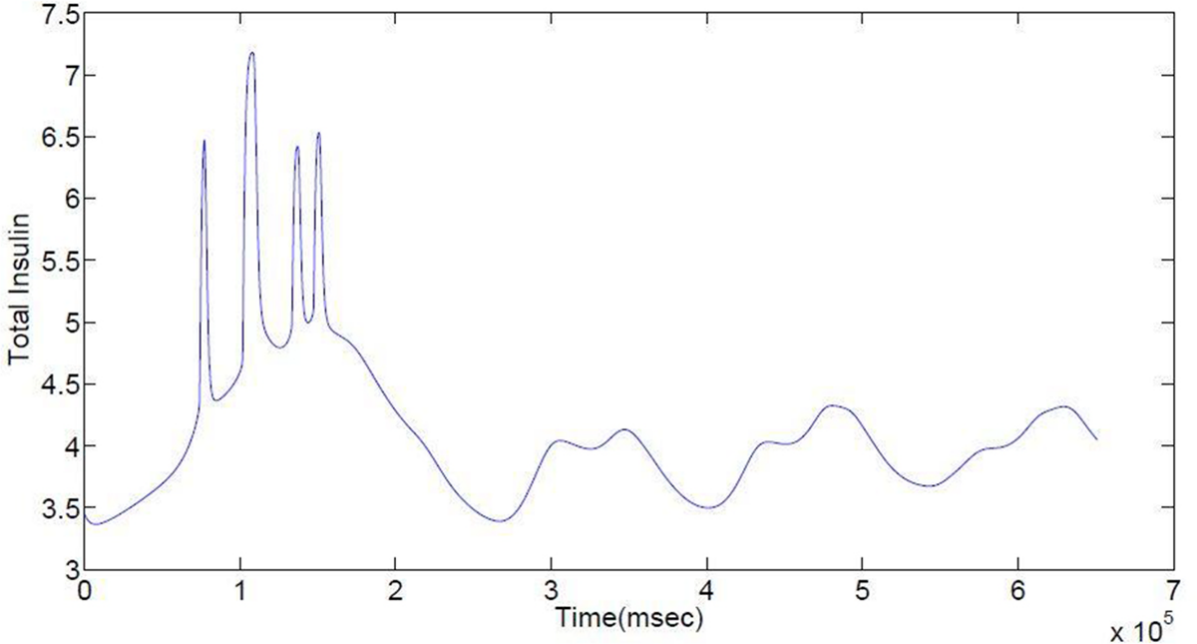
**Insulin secretion failure of the eight cells system**. There are three unhealthy cells uniformly distributed in the 2 X 2 X 2 topology

To study possible reasons for the failure of insulin secretion caused by unhealthy cells, consider a 2 × 2 × 2 model of eight cells, in which three unhealthy cells are uniformly distributed. Figures in Additional file 4 show the behaviors of all the variables in this model for each of the eight cells in each figure. The insulin secretion starts to fail at around time 2 × 10^5 ^ms as shown in Figure 8. Except for the plot of [G6P] (see Additional file 4: Figure 10), it is hard to distinguish healthy cells from unhealthy cells in all the plots, such as that of membrane potential in Figure 5 of Additional file 4. In Figure 10 of Additional file 4 eight curves are partitioned into two groups. The upper group consists of healthy cells, while the lower one consists of unhealthy cells. These two groups of curves do not intersect with each other after the failure, but they are mixed together before the failure. This observation suggests that G6P might be closely related to the insulin secretion failure. In order to test this hypothesis, the two groups of curves were manually reset to see whether insulin secretion can be reinstated: if the insulin secretion fails for 5 × 10^5 ^ms, the values of [G6P] in all cells are reset to 279, the initial value of [G6P] used in the simulation. The oscillations of insulin secretion are resumed (see Additional file 5: Figures 1 and 2). The black vertical lines in these figures are the times when the values of [G6P] are equalized. The results show that the divergence of the two groups of [G6P] leads to the disappearance of insulin secretion oscillations.

Since an unhealthy cell has a smaller mitochondria/cytosol volume ratio than that of a healthy cell, unhealthy cells usually have lower ATP (from oxidative phosphorylation) levels. ATP has a negative feedback to phosphofructokinase (PFK) in glycolysis. When the ATP level is lower, the PFK reaction becomes faster, which directly consumes G6P faster leading to lower G6P levels in unhealthy cells than in healthy cells. As shown in Figure 10 of Additional file 4 the levels of G6P in healthy cells are higher than those in unhealthy cells. At the same time, because of the low production rate of ATP in unhealthy cells, the ratio of ATP to ADP gets lower. ATP-sensitive K^+ ^channels in the plasma membrane are activated by ADP and inactivated by ATP, so the ratio of these nucleotides determines the fraction of open ATP-sensitive K^+ ^channels. When the ATP/ADP ratio is low there is an increase in the number of open ATP-sensitive K^+ ^channels, which results in the difficulty of membrane depolarization. Voltage-dependent Ca^2+ ^channels are blocked. Since the insulin is secreted when Ca^2+ ^exceeds a certain level, the blocked Ca^2+ ^channels will reduce insulin secretion. Therefore, raising the levels of G6P in unhealthy cells back to normal will bring back normal insulin secretion. This analysis suggests the hypothesis that increasing the value of any substance ahead of the ATP synthesis in the pathway, such as the substances FBP and NADH in mitochondria, reinstates the oscillations. Manually resetting the values of either [FBP] or [NADH] in all the cells to the same initial value confirms the conjecture: not only can G6P restart the insulin oscillations, but also FBP and NADHm can restart the oscillations.

Since G6P and FBP are both substances in the glycolysis pathway, if the rate of glycolysis were faster, the oscillations of insulin might be resumed as well. In order to test this conjecture, the glucokinase level is raised fourfold after the insulin failure is detected. Glucokinase is the enzyme that phosphorylates glucose to glucose-6-phosphate (G6P). Figures 3 and 4 in Additional file 5 show that the oscillations of insulin are resumed when the glucokinase level increased. Two oscillation periods are shown in Figure 5 of Additional file 5 (from the earlier 125 cell model): The longer period (slow oscillations) is caused by the glycolytic oscillations, while Ca^2+ ^feedback is responsible for the fast oscillations. Only the short period remains in Figure 6 of Additional file 5. This observation suggests that if the longer period could be reestablished in Figure 6 of Additional file 5 the insulin oscillations might be reinstated. Since the glycolysis pathway is responsible for the slow oscillations, that pathway may need some stimulus. The simulation results in Figures 4 and 5 of Additional file 5 demonstrate that by increasing the glucokinase level in the glycolysis pathway, the insulin oscillations can be resumed after the failure.

**Figure 9:**
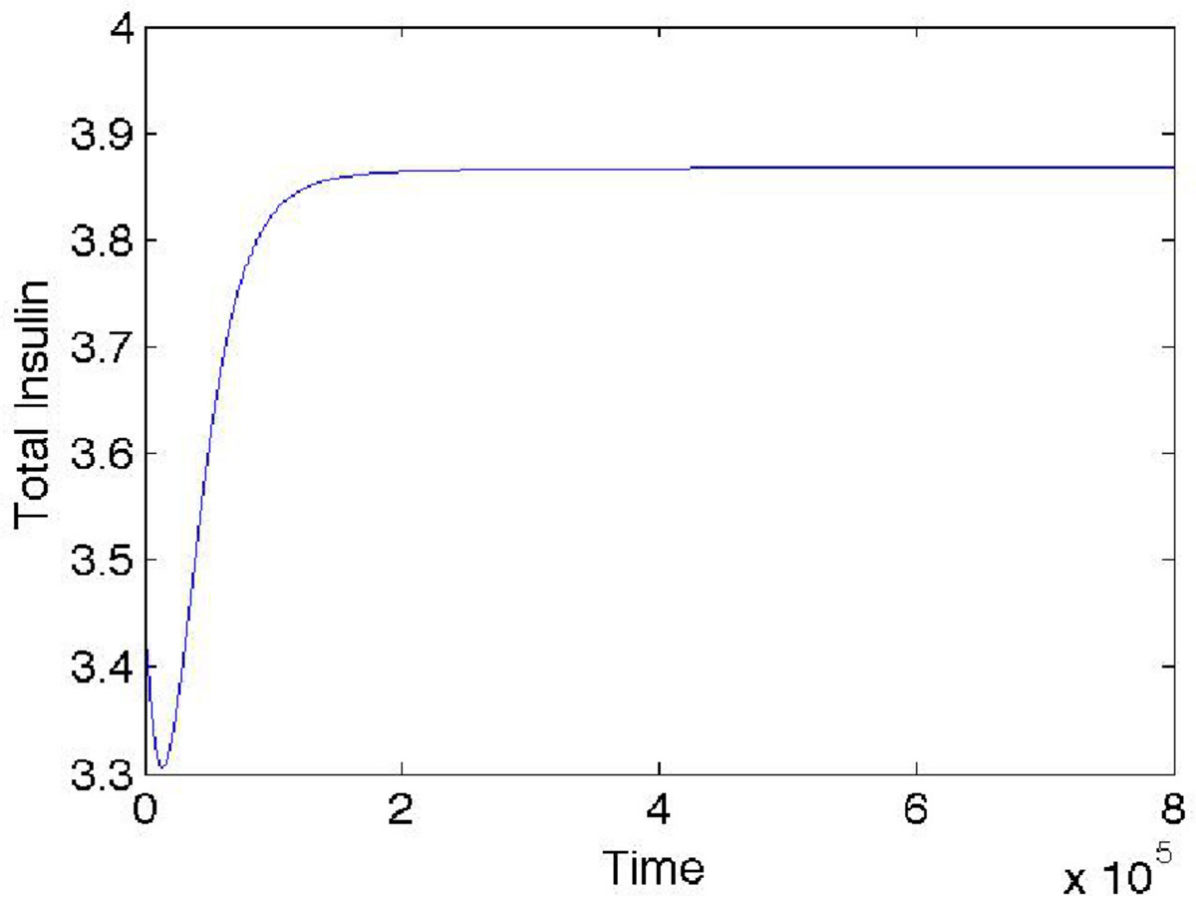
**Total insulin secretion of the eight unhealthy cells system with the stimulation on glucokinase of the cells**. All the cells are placed in the 2 × 2 × 2 topology

Figure 7 in Additional file 5 shows the simulation result when the three unhealthy cells are placed together in the 2 × 2 × 2 grid. With a high ratio of unhealthy cells to total cells (more than .20), the insulin secretion fails. If the glucokinase level in the unhealthy cells is raised fourfold and then kept unchanged during the simulation, the total insulin secretion becomes normal (see Additional file 5: Figure 8). From Figure 9 in Additional file 5 all the unhealthy cells look like the healthy cells. Even though those unhealthy cells are still unhealthy, if their glucokinase levels are high enough, the overall behavior of the system will be dominated by the healthy cells. If there is no healthy cell, only unhealthy cells with high glucokinase levels, the system can't be repaired, see Figure 9. Therefore, the unhealthy cells are rendered harmless by high glucokinase level and other healthy cells in the network.

## Conclusion

In conclusion, the insulin secretion of pancreatic islets usually will be functionally destroyed when there are more than 20% of cells being unhealthy among all cells. The more unhealthy cells there are, the more irregular insulin secretion will be. Increasing the level of glucokinase can make the pancreatic islet function normally when there is a high fraction of unhealthy cells by increasing the glucose absorption of the glycolysis pathway. This has implications for the clinical treatment of type II diabetes. Currently there are three classes of medications used to treat type II diabetes. The first treatment is to increase the amount of insulin secreted by the pancreas by inhibiting the opened delayed rectifying K^+ ^channels. The second treatment is to increase the sensitivity of target organs to insulin. The third treatment is to decrease the rate at which glucose is absorbed from the gastrointestinal tact, which is a method to reduce the glucose uptake from food. It appears that all the available treatments are insufficient to stem the tide. Therefore, new treatments are currently under investigation including the development of therapeutic agents with novel action mechanisms. Recently, researchers have identified glucokinase as an outstanding drug target for developing antidiabetic medicines [25-30]. Assuming the mathematical models are valid, the simulation results demonstrate that stimulating glucokinase can make unhealthy pancreatic islets function normally, consistent with new antidiabetic medicines. Such multiple cell models are good candidates for guiding the development of the next generation of antidiabetic medicines.

## Abbreviations

ADP: adenosine diphosphate; ATP: adenosine triphosphate; ER: endoplasmic reticulum; F6P: fructose-6-phosphate; FADH: flavin adenine dinucleotide; FBP: fructose-1,6-bisphosphate; G6P: glucose 6-phosphate; GPDH: glyceradehyde 3-P dehydrogenase; NADH: nicotinamide adenine dinucleotide plus hydrogen; PDH: pyruvate dehydrogenase; PKF: phosphofructokinase; PTP: permeability transition pore; TCA: tricarboxylic acid.

## Competing interests

The authors declare that they have no conflict of interest.

## Authors' contributions

YP, DCS, LTW and YC designed the model. DCS provided the biology knowledge. SL and DCS identified the parameters to be modified for unhealthy cells. YP wrote the code and did the simulations. YP, DCS, LTW and YC analyzed data. YP drafted the manuscript. LTW, YC made critical revisions to the manuscript. All authors have read and approved the final manuscript.

## Supplementary Material

Additional file 1 **Cell membrane potential, calcium and insulin oscillation plots illustrating the different burst domain**. Contains cell membrane potential, calcium and insulin oscillation plots illustrating the periodic bursting domain, burst formation domain, burst loss domain and decoupling domain. Click here for file

Additional file 2 **Multiple cells simulation**. The simulation results of 125 cells and one thousand cells with different percentages of unhealthy cells. Click here for file

Additional file 3 **Simplified multiple cells simulation**. The simulation results of the simplified multiple cells model. Click here for file

Additional file 4 **The behaviors of all the variables in eight cells model**. Figures that show the behaviors of all the variables in eight cells model for each of the eight cells. Click here for file

Additional file 5 **Reinstate oscillations of insulin secretion**. Figures that demonstrate the reinstatement of insulin secretion by increasing level of G6P and glucokinase. Click here for file

## Declarations

The publication costs for this article were funded by the corresponding author with the above sources of support.

This article has been published as part of *BMC Medical Genomics *Volume 6 Supplement 3, 2013: Selected articles from the IEEE International Conference on Bioinformatics and Biomedicine 2012: Medical Genomics. The full contents of the supplement are available online at http://www.biomedcentral.com/bmcmedgenomics/supplements/6/S3.
