# Supplementary material for: The effect of unhealthy β-cells on insulin secretion in pancreatic islets
Source: BMC Med Genomics. 2013 Nov 11;6(Suppl 3):S6. doi: 10.1186/1755-8794-6-S3-S6 (PMC3981690; doi:10.1186/1755-8794-6-S3-S6)
Supplement: Additional file 1 — Cell membrane potential, calcium and insulin oscillation plots illustrating the different burst domain. Contains cell membrane potential, calcium and insulin oscillation plots illustrating the periodic bursting domain, burst formation domain, burst loss domain and decoupling domain. [file 1755-8794-6-S3-S6-S1.pdf]

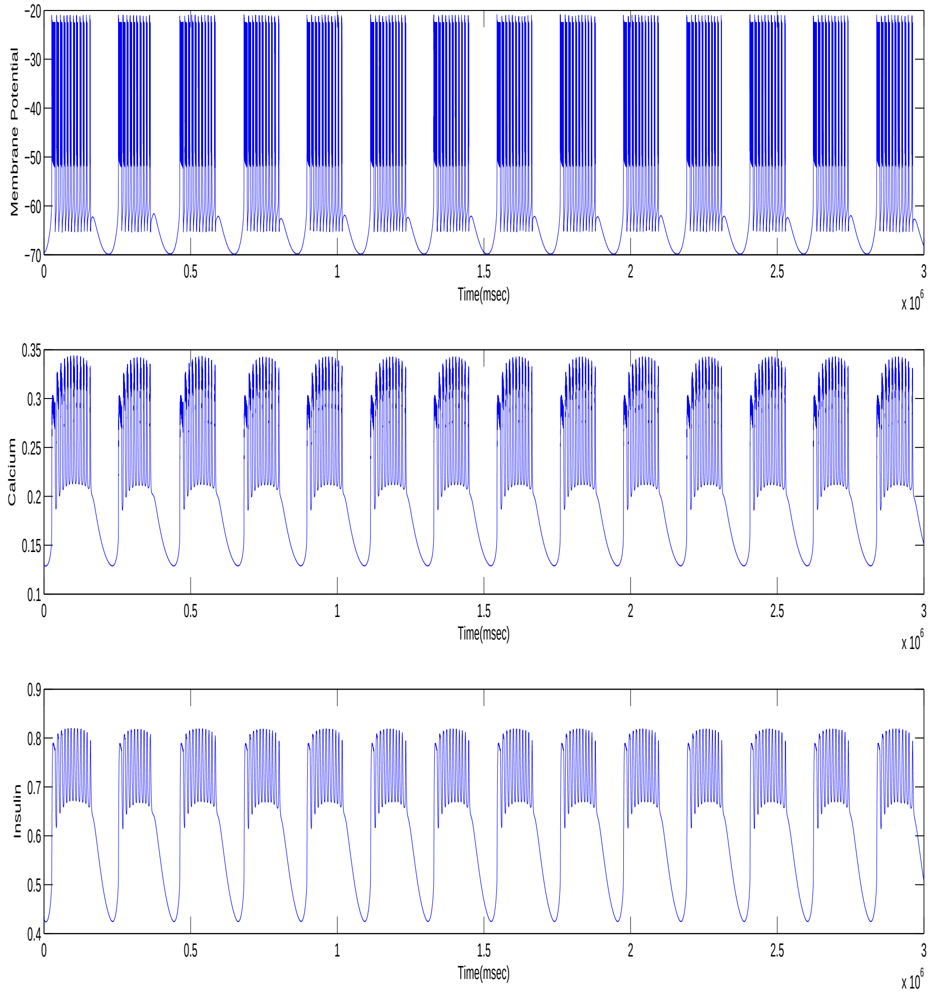

Figure 1: Cell membrane potential, calcium and insulin oscillation plots illustrating the periodic bursting domain (functional mitochondrial volume/ cytoplasmic volume = 7.3%).

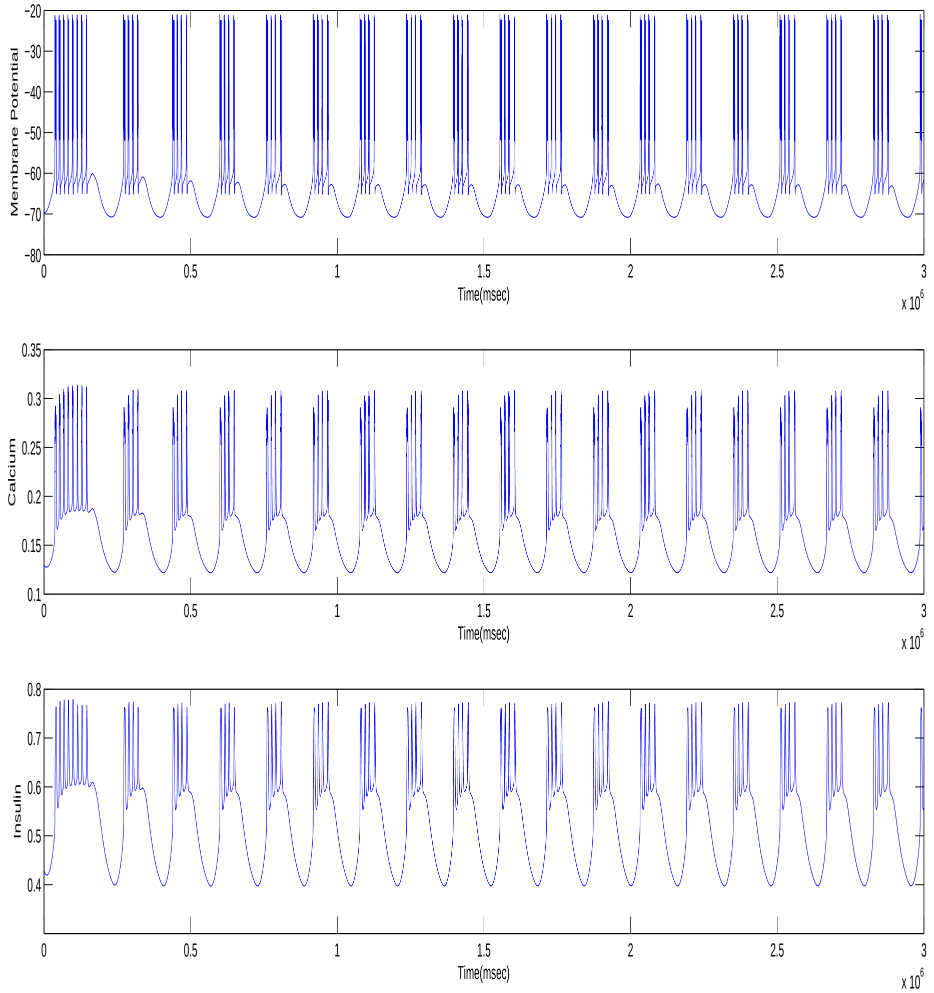

Figure 2: Cell membrane potential, calcium and insulin oscillation plots illustrating the burst formation domain (functional mitochondrial volume/ cytoplasmic volume = 6.21%, corresponding to 85% of the normal value used in Figure 1).

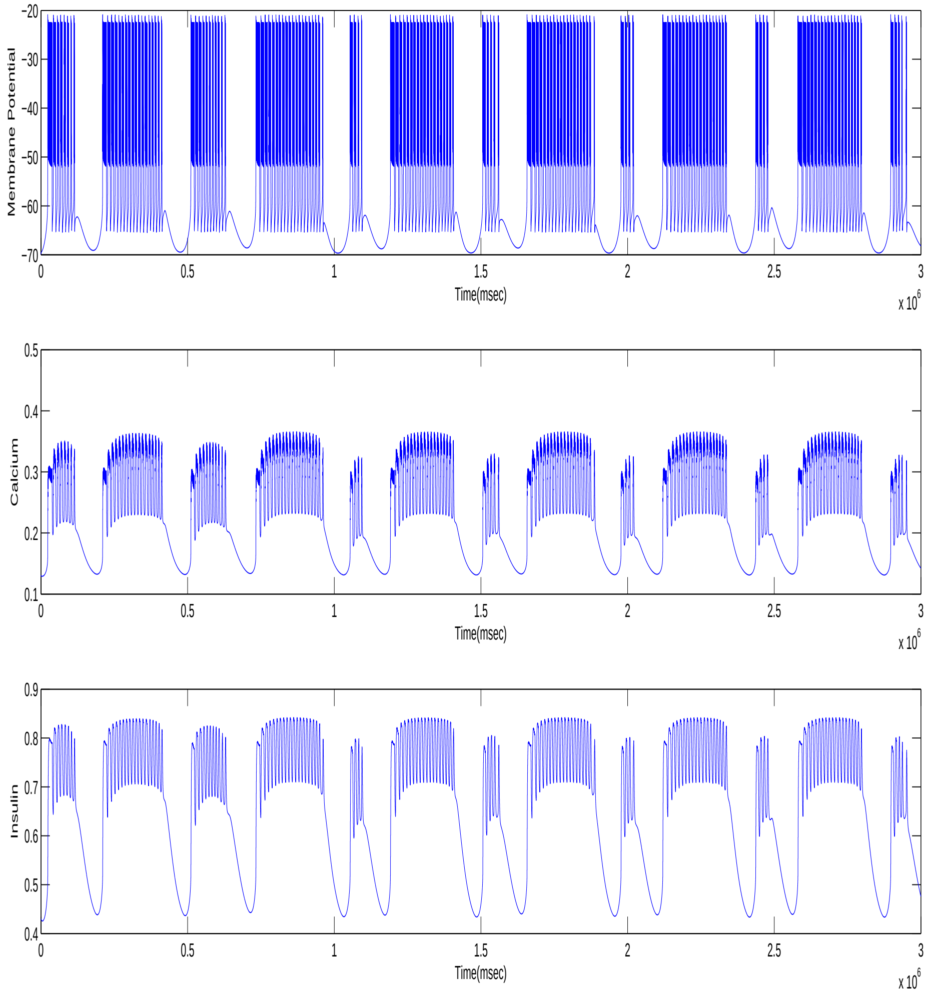

Figure 3: Cell membrane potential, calcium and insulin oscillation plots illustrating the burst loss domain (functional mitochondrial volume/ cytoplasmic volume = 8.06%, corresponding to 110% of the normal value used in Figure 1).

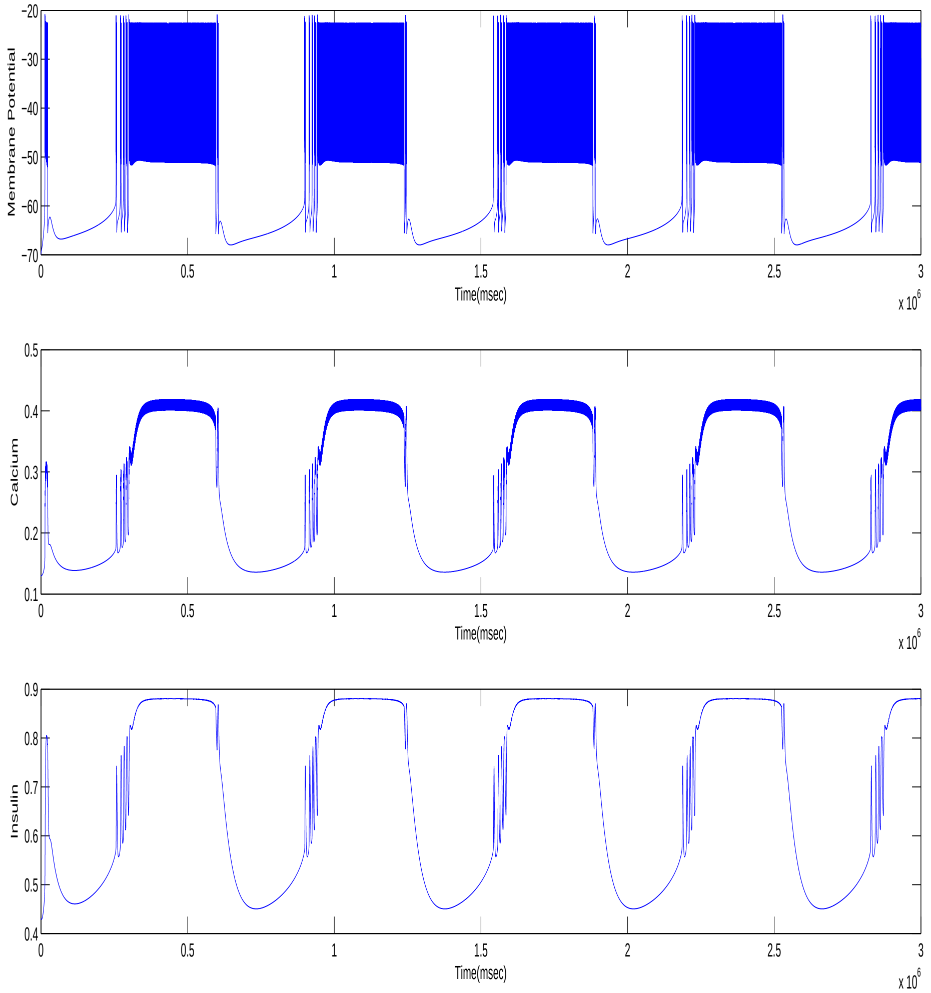

Figure 4: Cell membrane potential, calcium and insulin oscillation plots illustrating the decoupling domain (functional mitochondrial volume/ cytoplasmic volume = 11.7%, corresponding to 160% of the normal value used in Figure 1.
