# Supplementary material for: The effect of unhealthy β-cells on insulin secretion in pancreatic islets
Source: BMC Med Genomics. 2013 Nov 11;6(Suppl 3):S6. doi: 10.1186/1755-8794-6-S3-S6 (PMC3981690; doi:10.1186/1755-8794-6-S3-S6)
Supplement: Additional file 2 — Multiple cells simulation. The simulation results of 125 cells and one thousand cells with different percentages of unhealthy cells. [file 1755-8794-6-S3-S6-S2.pdf]

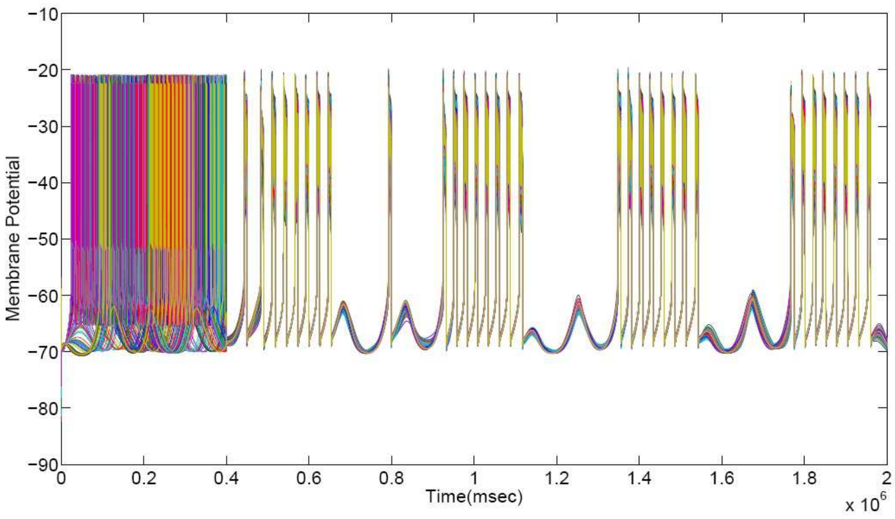

Figure 1: Membrane potential of 125 healthy cells.

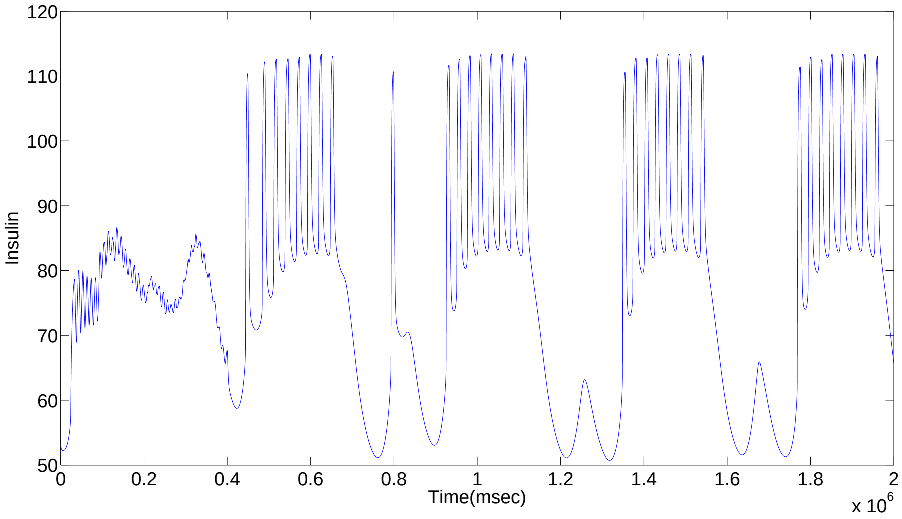

Figure 2: Insulin secretion of 125 healthy cells.

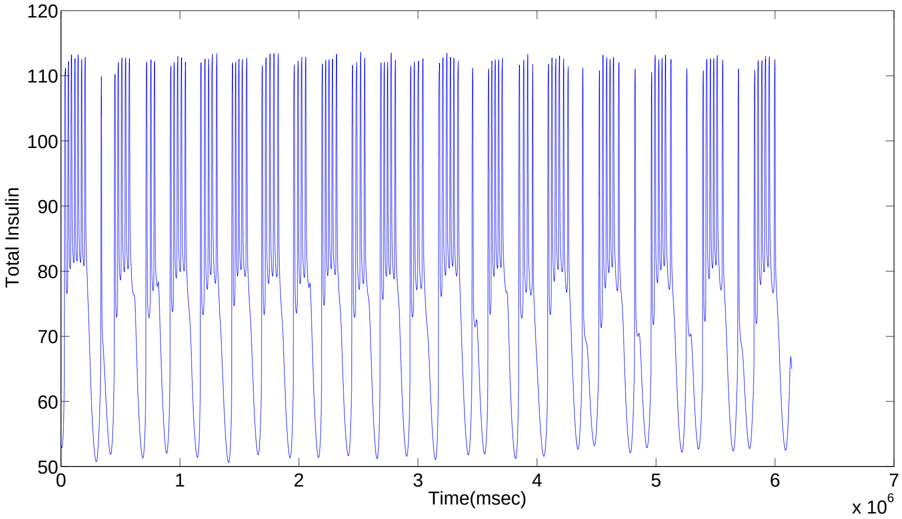

Figure 3: Insulin secretion of 125 total cells with 10% unhealthy cells.

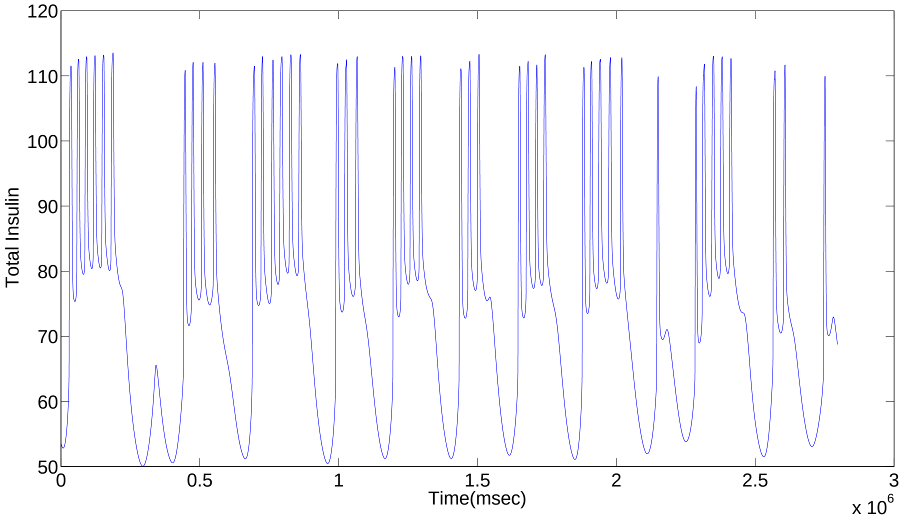

Figure 4: Insulin secretion of 125 total Cells with 15% unhealthy cells.

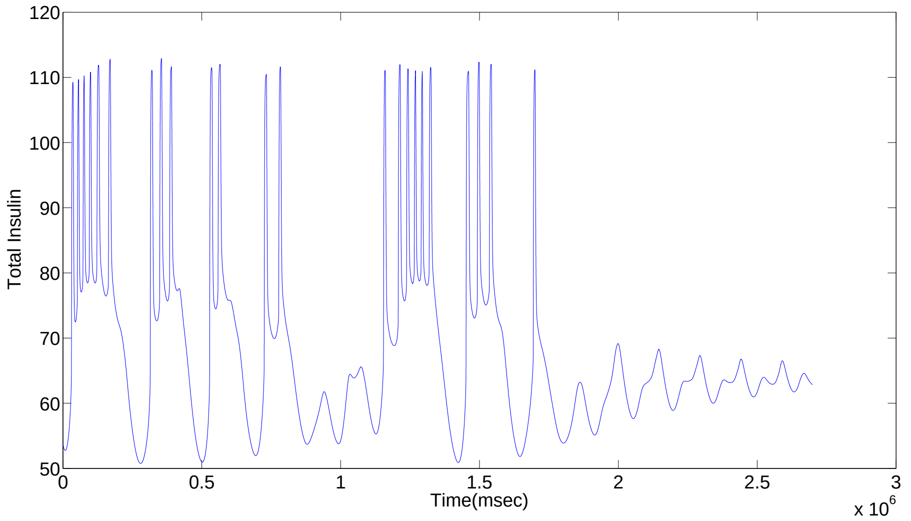

Figure 5: Insulin secretion of 125 total Cells with 20% unhealthy cells.

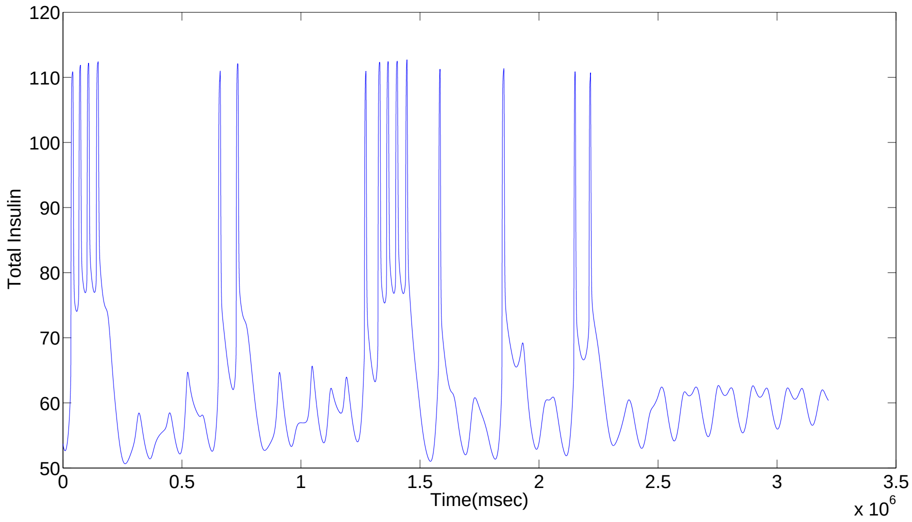

Figure 6: Insulin secretion of 125 total Cells with 30% unhealthy cells.

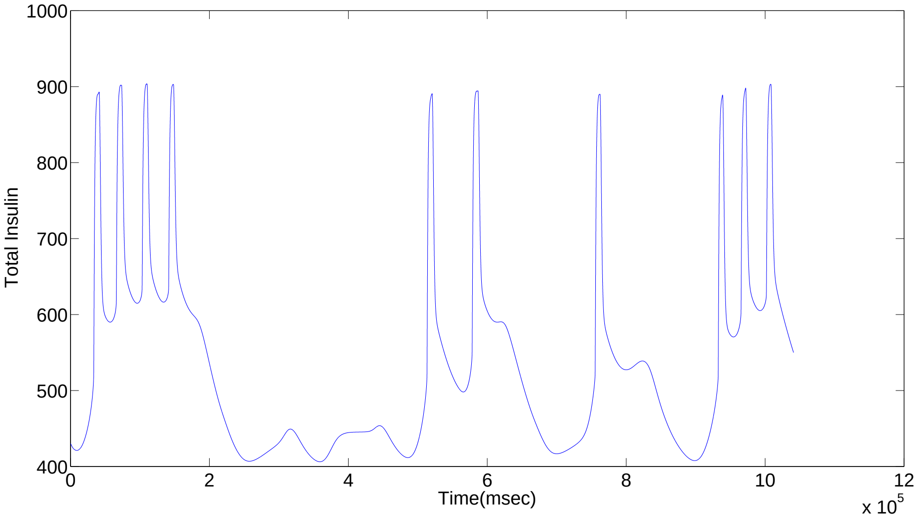

Figure 7: One thousand cells with 30% unhealthy cells.

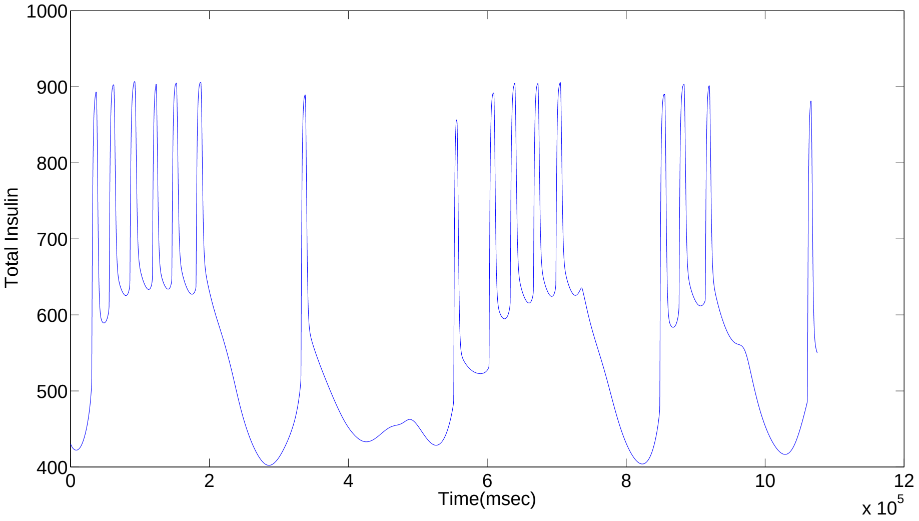

Figure 8: One thousand cells with 20% unhealthy cells.

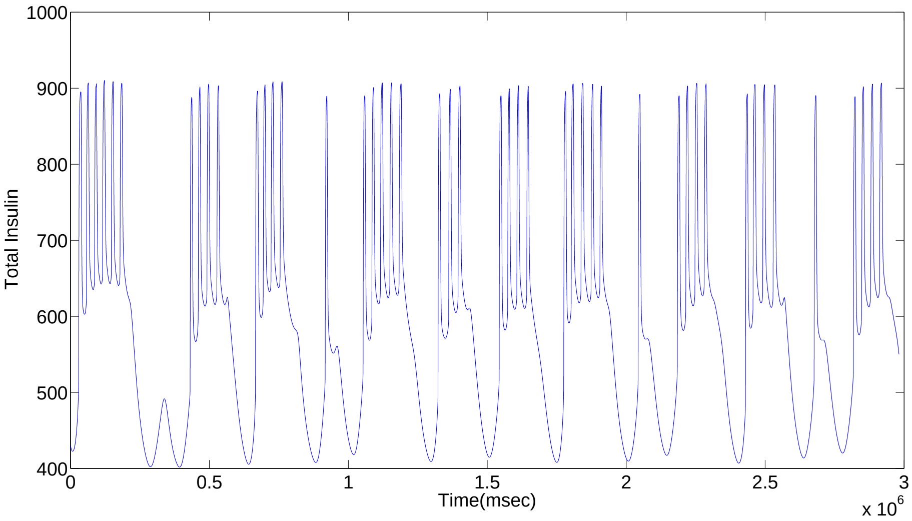

Figure 9: One thousand cells with 15% unhealthy cells.
