# Supplementary material for: The effect of unhealthy β-cells on insulin secretion in pancreatic islets
Source: BMC Med Genomics. 2013 Nov 11;6(Suppl 3):S6. doi: 10.1186/1755-8794-6-S3-S6 (PMC3981690; doi:10.1186/1755-8794-6-S3-S6)
Supplement: Additional file 3 — Simplified multiple cells simulation. The simulation results of the simplified multiple cells model. [file 1755-8794-6-S3-S6-S3.pdf]

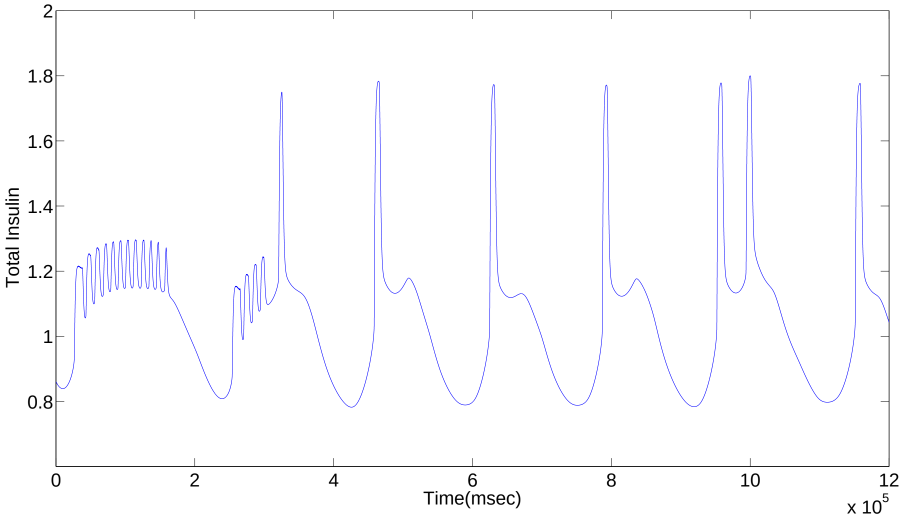

Figure 1: Total insulin of one bad cell and one good cell.

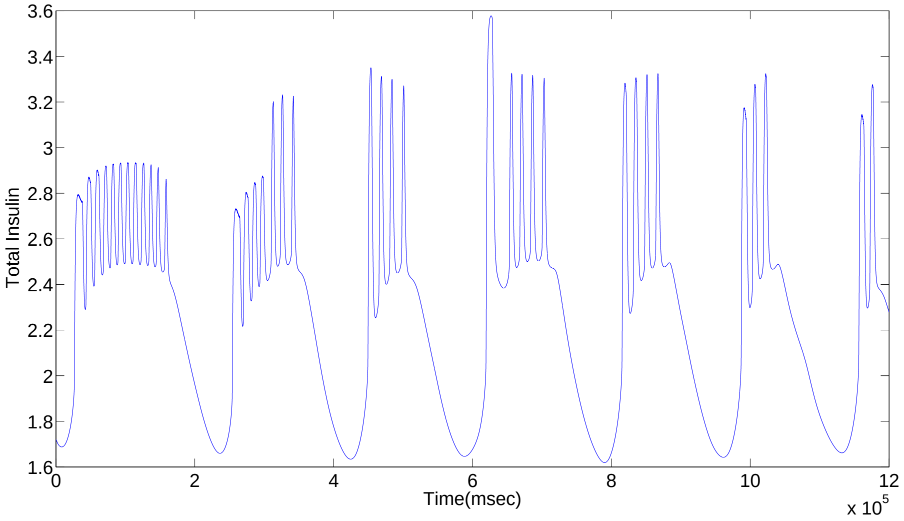

Figure 2: Total insulin of one bad cell and three good cells.
