# Supplementary material for: The effect of unhealthy β-cells on insulin secretion in pancreatic islets
Source: BMC Med Genomics. 2013 Nov 11;6(Suppl 3):S6. doi: 10.1186/1755-8794-6-S3-S6 (PMC3981690; doi:10.1186/1755-8794-6-S3-S6)
Supplement: Additional file 4 — The behaviors of all the variables in eight cells model. Figures that show the behaviors of all the variables in eight cells model for each of the eight cells. [file 1755-8794-6-S3-S6-S4.pdf]

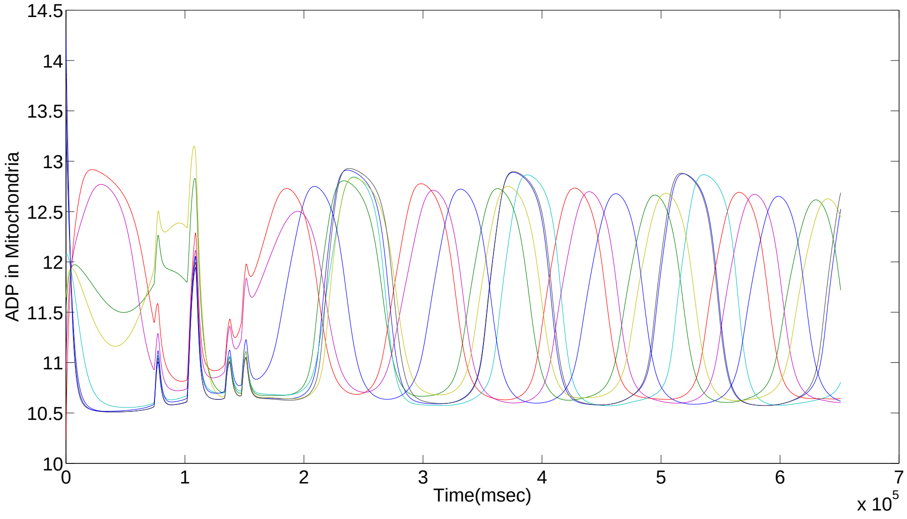

Figure 1: ADP in the mitochondria.

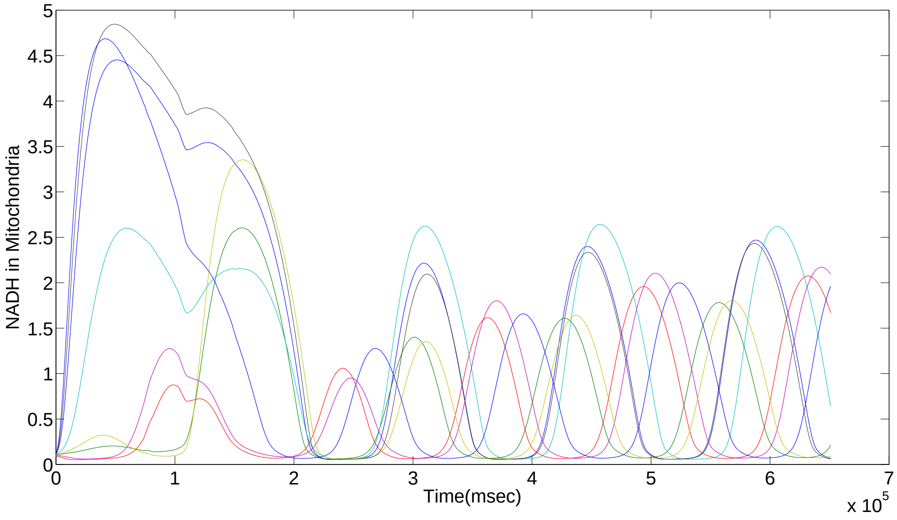

Figure 2: NADH in the mitochondria.

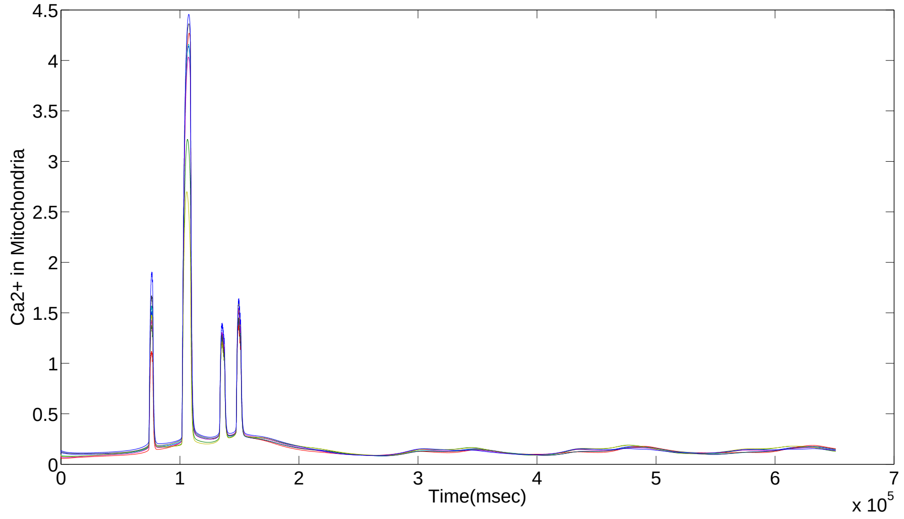

Figure 3: Calcium ion in the mitochondria.

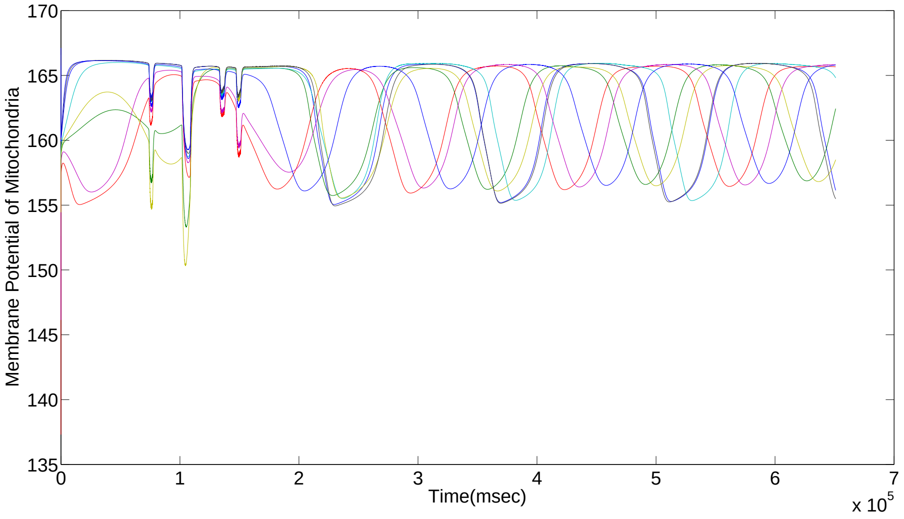

Figure 4: Membrane potential of mitochondria.

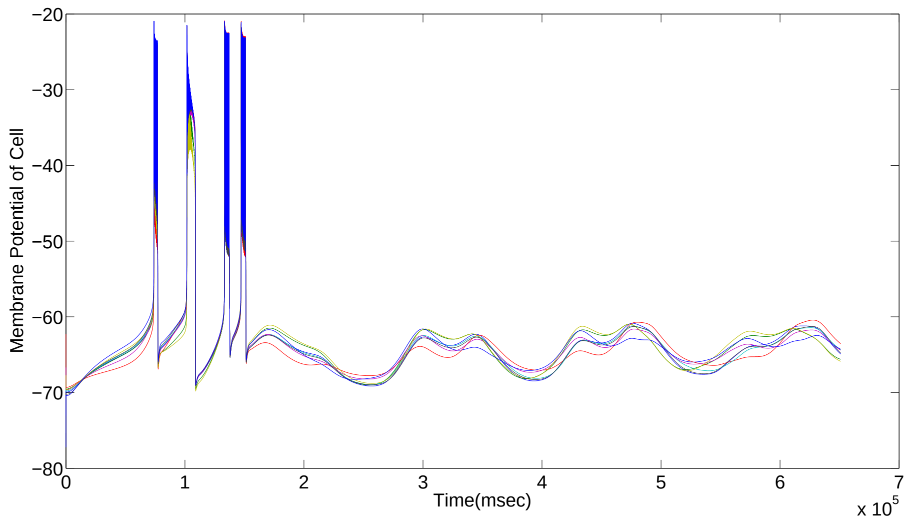

Figure 5: Membrane potential of cell.

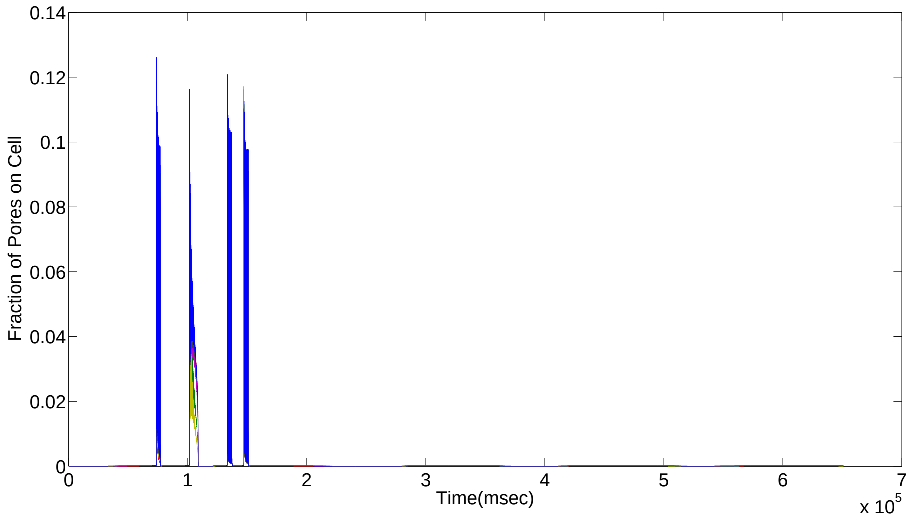

Figure 6: Fraction of open pores on cell membrane.

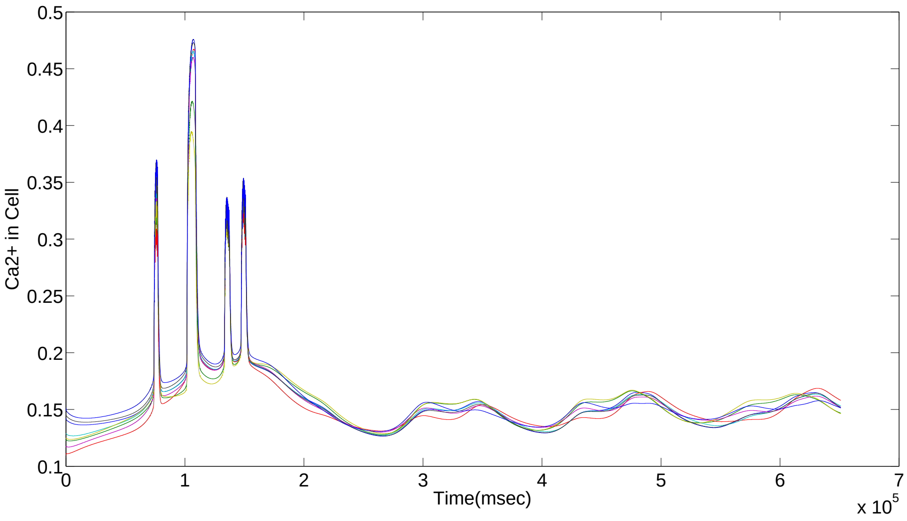

Figure 7: Calcium ion in the cell.

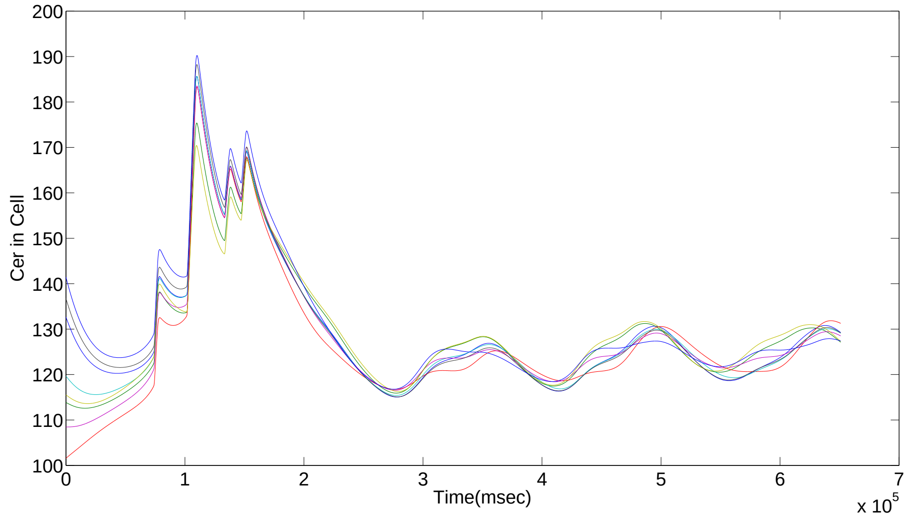

Figure 8: Cer in the cell.

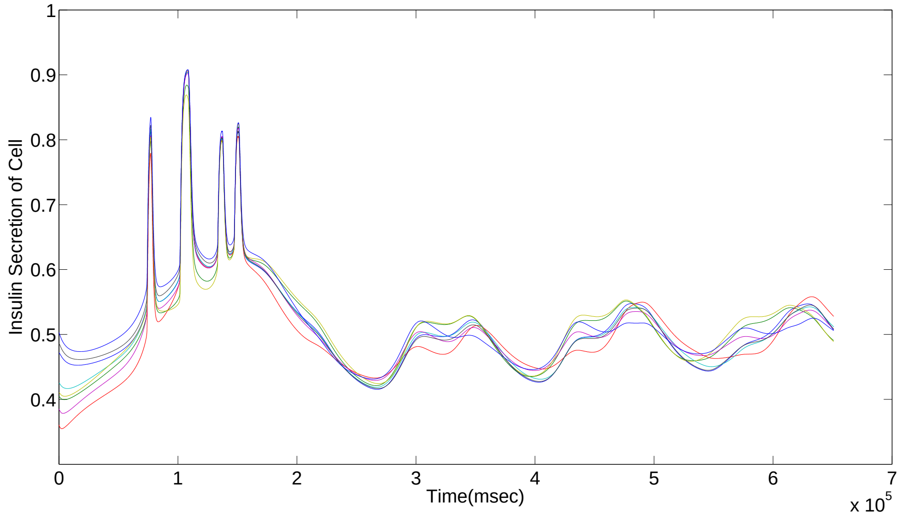

Figure 9: Insulin secretion of the cell.

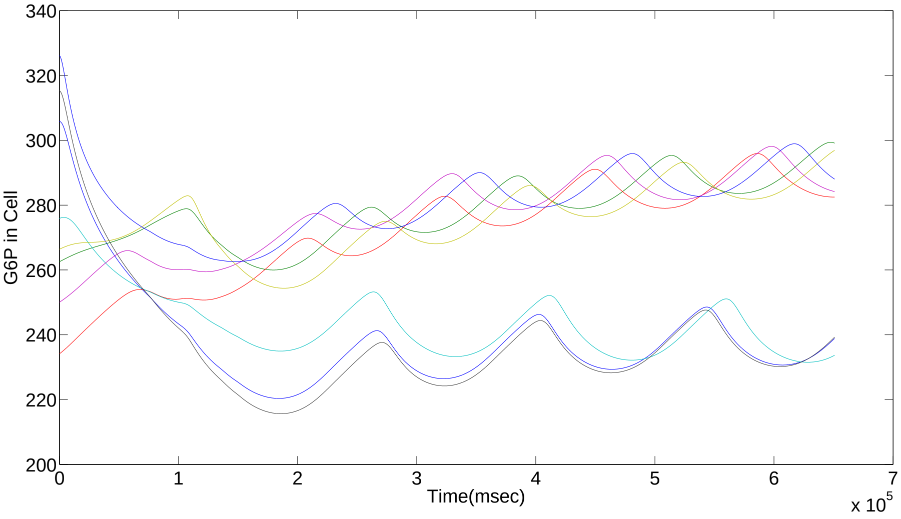

Figure 10: G6P in the cell.

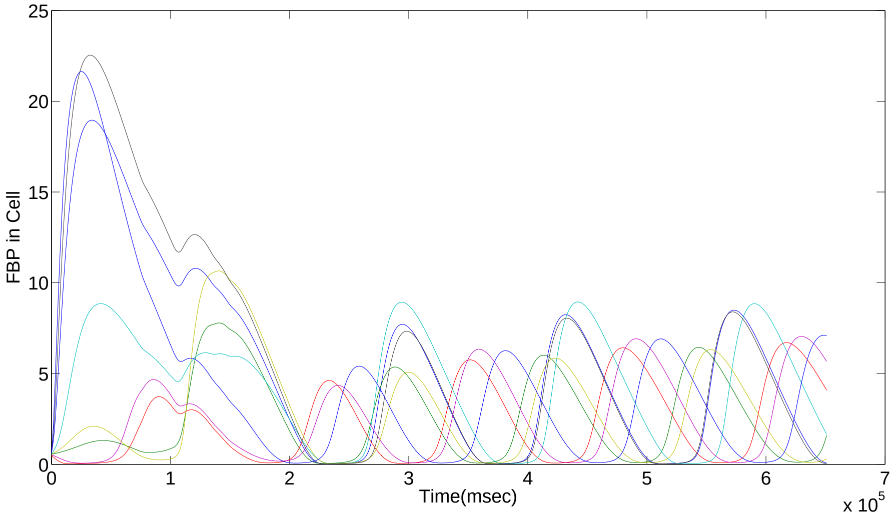

Figure 11: FBP in the cell.

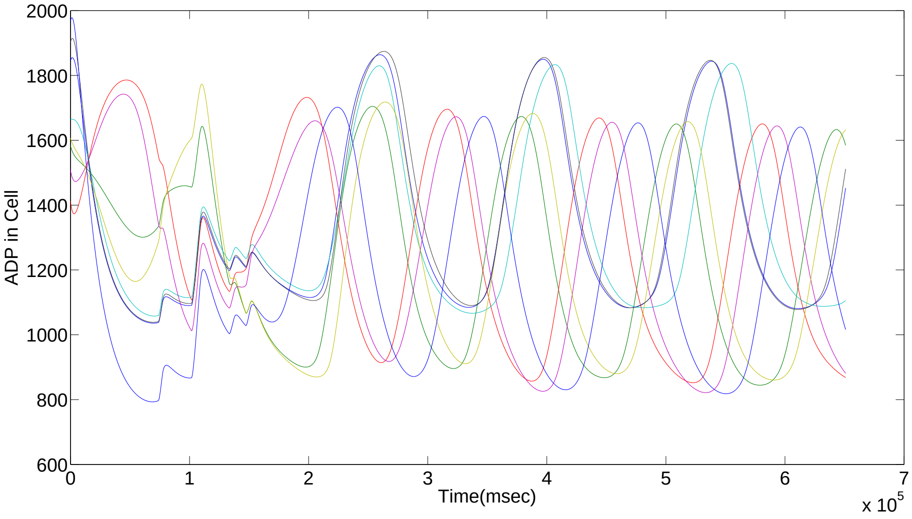

Figure 12: ADP in the cell.
