# Supplementary material for: The effect of unhealthy β-cells on insulin secretion in pancreatic islets
Source: BMC Med Genomics. 2013 Nov 11;6(Suppl 3):S6. doi: 10.1186/1755-8794-6-S3-S6 (PMC3981690; doi:10.1186/1755-8794-6-S3-S6)
Supplement: Additional file 5 — Reinstate oscillations of insulin secretion. Figures that demonstrate the reinstatement of insulin secretion by increasing level of G6P and glucokinase. [file 1755-8794-6-S3-S6-S5.pdf]

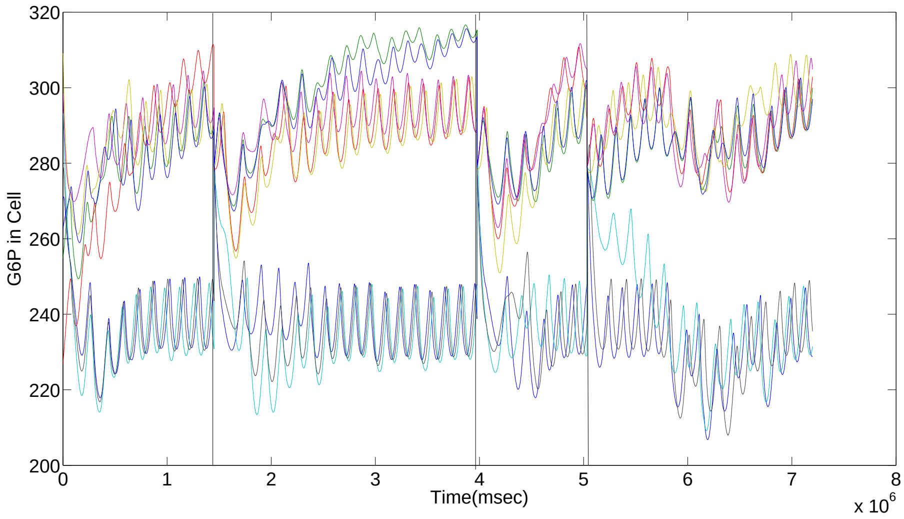


Figure 1: Drag G6P to be equal to reinstate oscillations of insulin secretion.

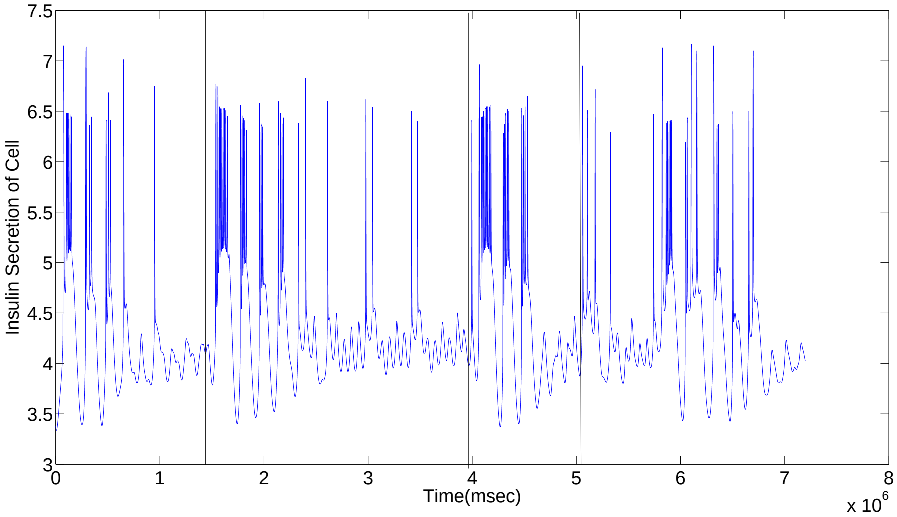


Figure 2: Reinstate oscillations of insulin secretion by increase level of G6P.

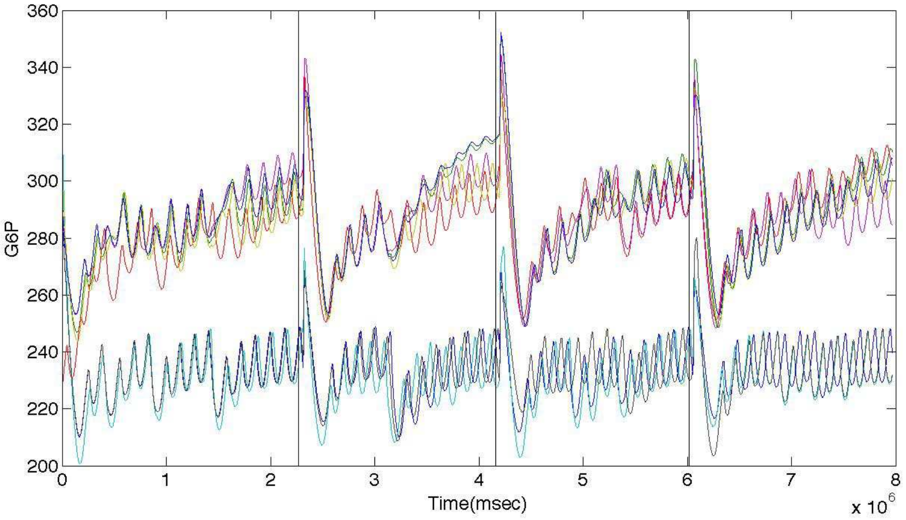


Figure 3: Increase Jgk to reinstate oscillations of insulin secretion.

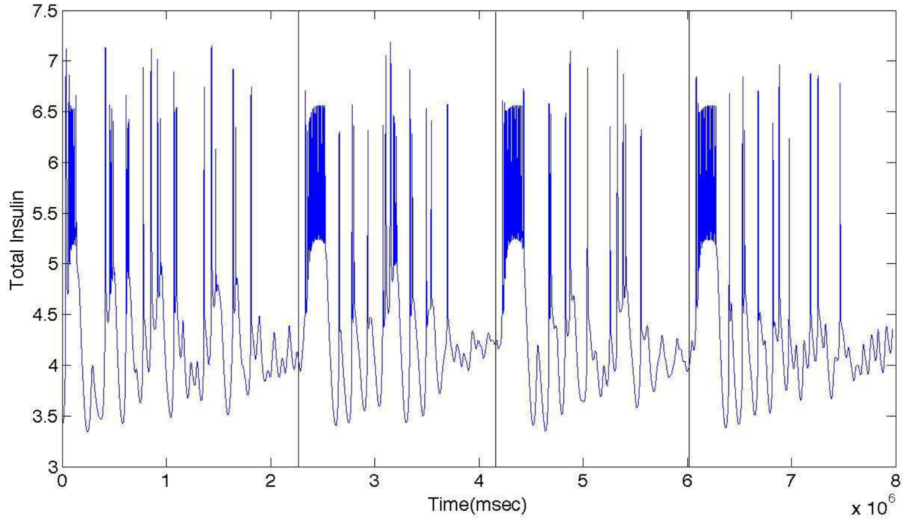


Figure 4: Reinstate oscillations of insulin secretion by increase glucokinase.

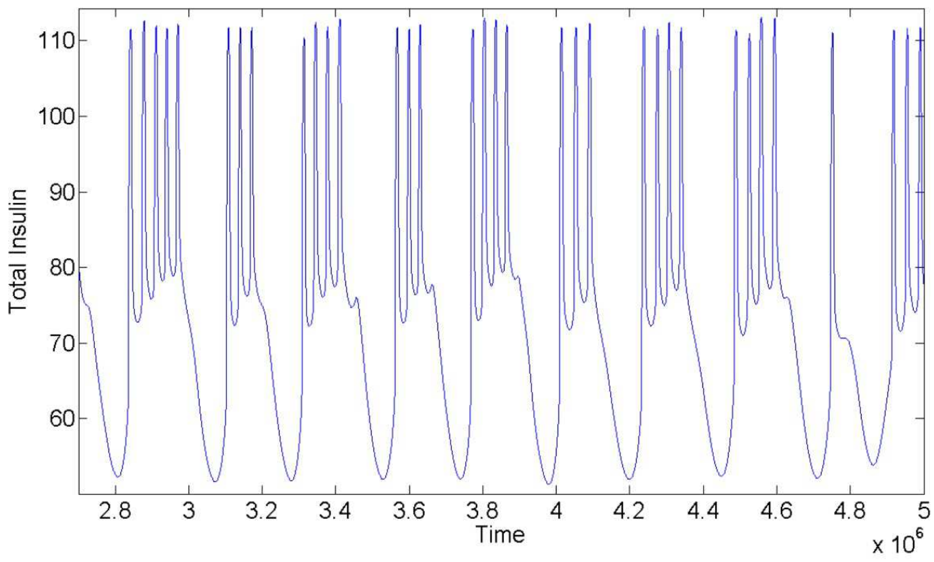


Figure 5: Zoomed figure of insulin secretion before failure.

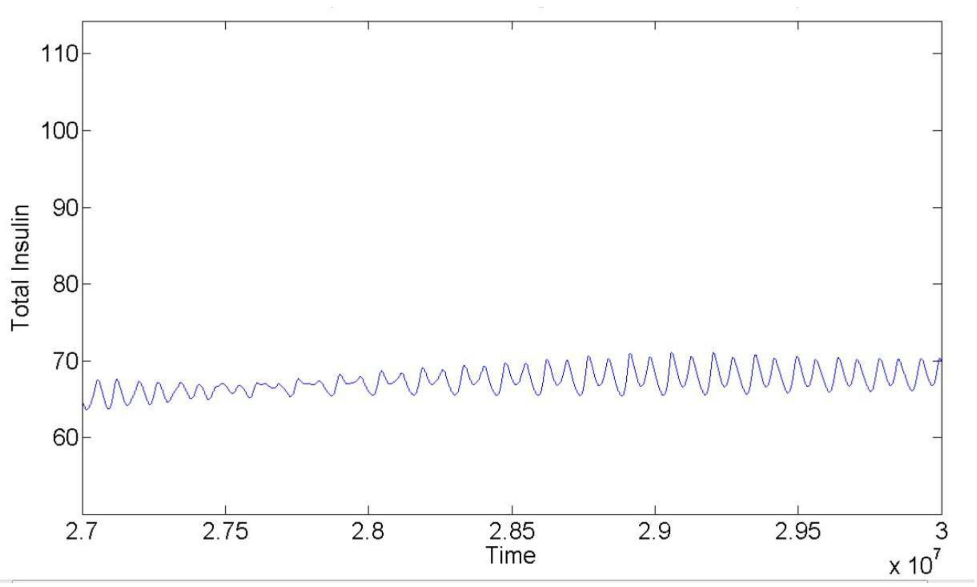


Figure 6: Zoomed figure of insulin secretion after failure.

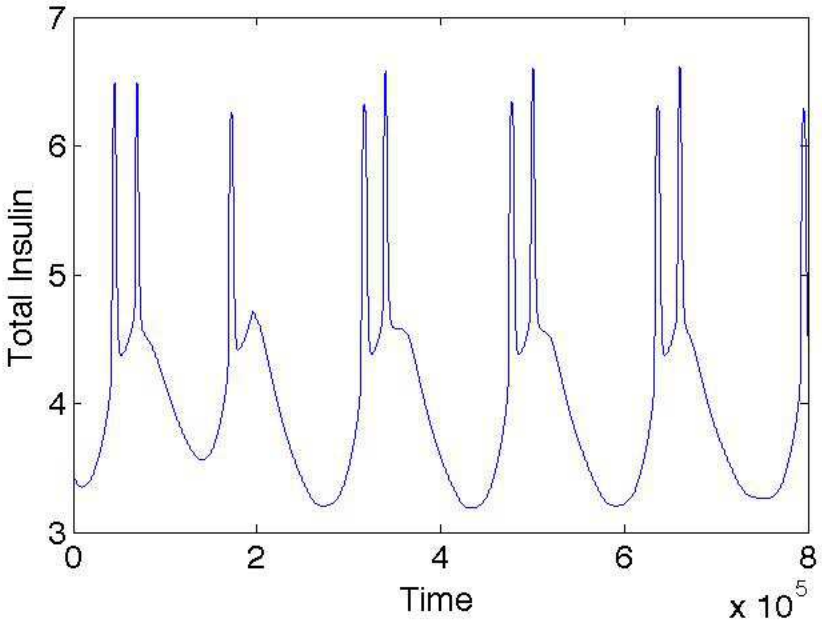


Figure 7: Total insulin of three unhealthy cells coupled with five healthy cells.

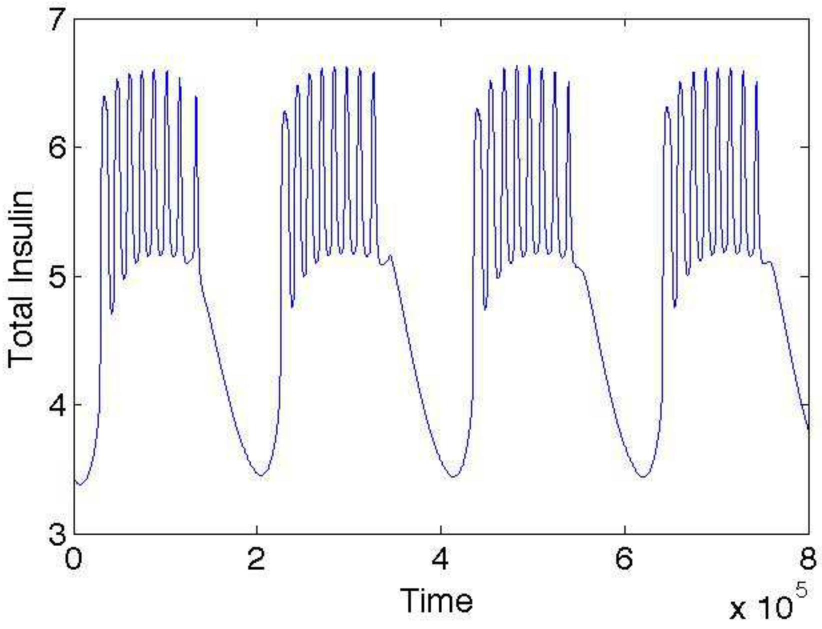


Figure 8: Total insulin secretion of eight healthy-unhealthy cells system with stimulation on glucokinase of unhealthy cells.

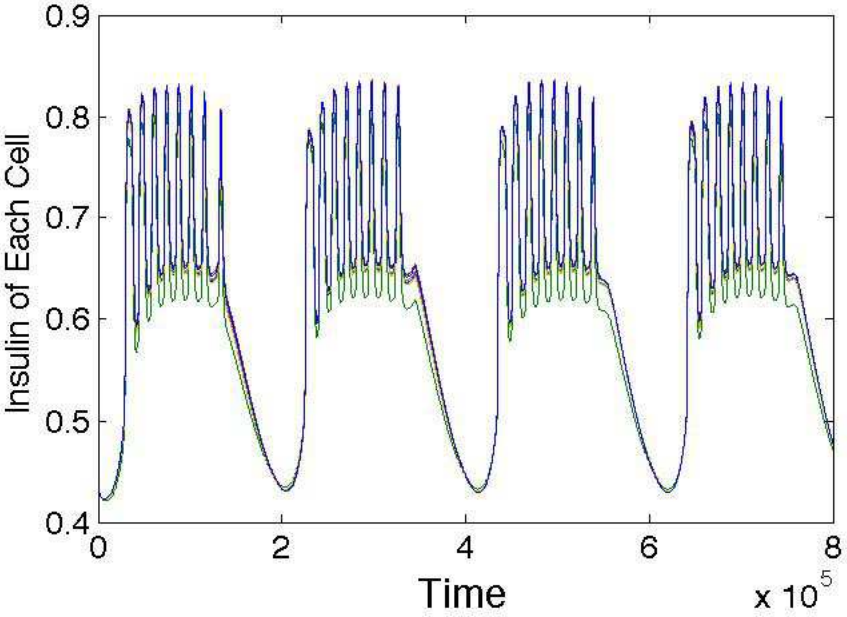


Figure 9: Individual insulin secretion of each cell in eight healthy-unhealthy cells system with stimulation on glucokinase of unhealthy cells.
